# Comparative genomics of endemic *Staphylococcus aureus* ST1 in New Zealand

**DOI:** 10.1128/msphere.00376-25

**Published:** 2025-09-23

**Authors:** Emma M. Voss, Gregory M. Cook, Christine Couldrey, Scott A. Ferguson, Chad Harland, Ali Karkaba, Scott McDougall, Sergio E. Morales, Jack Rolfe, James E. Ussher, Rhys T. White, Liam Williams, John Williamson

**Affiliations:** 1 Research and Development, Livestock Improvement Corporation (LIC), Newstead, New Zealand; 2 Department of Microbiology and Immunology, University of Otago 2495, Dunedin, New Zealand; 3 Queensland University of Technology, School of Biomedical Sciences 588731, Brisbane, Queensland, Australia; 4 Cognosco, Anexa Veterinary Services, Morrinsville, New Zealand; 5 American International University 589384, Jahra, Kuwait; 6 MPG Ranch, Missoula, Montana, USA; 7 Diagnostics, Livestock Improvement Corporation (LIC), Riverlea, New Zealand; 8 Health Security, New Zealand Institute for Public Health and Forensic Science, Porirua, New Zealand; University of Nebraska Medical Center College of Medicine, Omaha, Nebraska, USA

**Keywords:** *Staphylococcus aureus*, genomics, antimicrobial resistance, phylodynamics, host adaptation, New Zealand

## Abstract

*Staphylococcus aureus* is a major zoonotic bacterial pathogen that causes a broad spectrum of human and animal diseases, including skin infections, sepsis, endocarditis, and bovine mastitis. In the geographically isolated setting of New Zealand, the population structure of *S. aureus* exhibits a distinct genomic profile. Globally, bovine *S. aureus* isolates are primarily associated with sequence types (STs), ST97 and ST151, whereas in New Zealand, ST1 predominates, accounting for approximately 70% of bovine isolates. ST1 is also a clinically significant sequence type in humans. This study employed a comparative One Health approach to investigate genetic differences in 520 *S. aureus* ST1 isolated from bovine milk and human clinical samples. We aimed to explore genomic features associated with persistence and diversification across hosts, focusing on antimicrobial resistance (AMR), virulence, and mobile genetic elements. Comparative genomics revealed that human isolates carried a significantly higher burden of AMR genes, consistent with clinical selective pressure. In contrast, 83% of bovine isolates harbored a prophage (φSabovST1) similar to *S. aureus* prophage φSaov3. This prophage encodes bovine-adapted leukocidins (LukMF’), supporting host-specific adaptation. Phylogenetic analysis revealed long branches, suggesting insufficient sampling, highlighting the need for broader genomic surveillance to resolve evolutionary relationships and transmission dynamics of *S. aureus* ST1 in New Zealand. These findings highlight the complex history of host interactions, historical transmission events, and ongoing bacterial adaptation. Expanding sampling efforts across human, animal, and environmental reservoirs will provide deeper insights into strain diversity, elucidate transmission pathways, and inform strategies to mitigate zoonotic risks.

**IMPORTANCE:**

This study presents a comprehensive genomic analysis of *S. aureus* ST1, a lineage that is unusually dominant in both bovine and human populations in New Zealand. Leveraging New Zealand’s geographical isolation, we provide critical insights into the persistence, diversification, and adaptation of *S. aureus*, offering valuable knowledge to advance disease prevention in both public and veterinary health and strengthening global biosecurity. The development of the first bovine ST1 reference genome serves as a valuable resource for future research, while the identification of a novel prophage (φSabovST1) carrying bovine-specific leukocidins underscores the role of mobile genetic elements in host specificity and virulence. Human isolates exhibited a higher prevalence of antimicrobial resistance genes. Phylogenetic analysis further revealed two main circulating clades of ST1 with interspersed host origins, highlighting the critical need for integrated One Health approaches to more effectively monitor and manage zoonotic pathogens across agricultural and public health systems.

## INTRODUCTION

*Staphylococcus aureus* is a commensal and opportunistic pathogen in humans and animals (1–5). In humans, 20–30% of individuals are colonized by *S. aureus*, ranging from asymptomatic carriage and mild skin infections to life-threatening conditions such as sepsis and endocarditis (2, 3, 5, 6). While humans are considered the primary reservoir, *S. aureus* is a significant pathogen in veterinary medicine, especially in bovine mastitis (1, 4, 7, 8). Bovine mastitis is an inflammatory disease of the mammary gland, primarily caused by bacterial infection, and remains one of the most economically impactful conditions in dairy cattle. It is responsible for global losses estimated at US $19.7–32 billion annually due to reduced milk production, discarded milk during the antibiotic withholding period, veterinary treatment, and the culling of affected animals (1, 7, 9, 10). *S. aureus* is a major causative agent of mastitis, leading to both subclinical and clinical intramammary infections and is often transmitted during milking (10–12).

The global population structure of *S. aureus* is shaped by host restriction, antimicrobial pressure, clonal expansion, and the movement of humans and animals (5, 13, 14). However, little is known about how these dynamics play out in isolated ecosystems like New Zealand, where strict biosecurity and geographical isolation provide a unique setting to study bacterial evolution (15). In population genetics, multilocus sequence typing (MLST) assigns sequence types (STs) based on housekeeping gene variations, while clonal complexes (CCs) group related strains (16–18). Globally, bovine-derived *S. aureus* isolates are predominantly associated with ST97/CC97 and ST151/CC151, both well adapted to cattle. However, in New Zealand, ST1/CC1, typically associated with humans, is most prevalent in the bovine-derived *S. aureus* population (7, 13, 14, 19, 20). This unusual distribution raises critical questions about host restriction, cross-species transmission, and zoonotic potential. Previous studies identified genomic similarities between human and bovine-derived ST1 isolates (21), suggested the possibility of frequent host jumps (13), and described a ruminant-host-adapted CC1 clade (22). However, ST1 evolutionary dynamics, gene flow mechanisms, and mobile genetic element (MGE) profiles in New Zealand remain poorly characterized. Given New Zealand’s heavy reliance on agriculture (23) and ST1’s zoonotic concerns, a deeper understanding of this lineage is essential for managing risks to both public and animal health. This study integrates time-calibrated phylogenetics, comparative resistome and virulome profiling, and MGE mapping to reconstruct ST1’s evolutionary history in New Zealand, specifically analyzing human and bovine-derived *S. aureus* ST1 isolates. By comparing genomic exchange and host divergence, our findings underscore the importance of adopting a One Health approach and highlight the need for coordinated surveillance of zoonotic pathogens across human and veterinary health systems.

## MATERIALS AND METHODS

### Bovine and human *S. aureus* isolates

As part of a nationwide New Zealand collection study (July 2019–November 2023), DairyNZ/Livestock Improvement Corporation (LIC) Taranaki trial (2005) and routine diagnostic work at Anexa Veterinary Services, *S. aureus* isolates (*n* = 928) were obtained from bulk tank milk (composite herd sample) (*n* = 618), foremilk aseptic quarter (individual animal sample from each quarter) (*n* = 305), and composite milk samples (combined quarters from an individual animal) (*n* = 5) across 15 regions. Samples were cultured on esculin sheep blood agar, and isolates were identified as *S. aureus* using coagulase, catalase, and hemolysis tests before being preserved in Laboratoire de Santé Publique du Québec (LSPQ) medium at −20°C. Full details are provided in the Supplemental Methods and Fig. S1.

Clinical human methicillin-susceptible *S. aureus* (MSSA) isolates (*n* = 126) were collected from soft tissue and wound infections at healthcare facilities in the Waikato, Lakes, and Bay of Plenty regions. Identification and susceptibility testing (European Committee on Antimicrobial Susceptibility Testing) were conducted using matrix-assisted laser desorption/ionization time-of-flight (Bruker, USA) and the BD Kiestra TLA system. Pathlab, a human pathology laboratory in New Zealand, processed these isolates. Isolates were initially stored on nutrient agar slopes and transported to the LIC Animal Health Laboratory in Hamilton, New Zealand. Thereafter, they were stored and processed using the same protocols as the bovine-derived *S. aureus* isolates.

### Genomic DNA extraction, sequencing, and analysis

*S. aureus* isolates (*n* = 1054) were re-streaked from LSPQ stocks, cultured on esculin sheep blood agar, and inoculated into tryptic soy broth. DNA was extracted using a Kingfisher machine (Thermo Fisher, New Zealand) and the BioSprint 96 DNA kit (Qiagen). Sequencing libraries were prepared in batches (see Supplemental methods), using NEBNext Ultra for Illumina sequencing on NovaSeq 6000 and X Plus (Annoroad Gene Technology Corporation, China) and the Illumina DNA Preparation Kit and Illumina UD Indexes for sequencing at LIC (Hamilton, New Zealand).

Quality control was performed using FastQC v.0.11.9 (24) and Nullarbor v.2.0 (https://github.com/tseemann/nullarbor, accessed 1 March 2022). For the initial species confirmation, lineage assignment, and quality filtering, bovine-derived *S. aureus* isolates were aligned to the bovine-associated reference genome *S. aureus* RF122 (GenBank: NC_007622), while human-derived *S. aureus* isolates were aligned to the human-associated reference genome *S. aureus* subsp. *aureus* MSSA476 (GenBank: NC_002953.3). This host-specific approach was used to improve alignment accuracy and ensure appropriate single-nucleotide polymorphism (SNP) calling during early analyses. Genomes with low sequencing depth (<50×), low N50 scores, or ≤70% *S*. *aureus* identity in Kraken2 (25) classification were excluded. Post-quality control (QC), 835 bovine and 122 human-derived *S. aureus* genomes remained (Tables S1 and S2). MLST and *spa* typing were conducted using MLST (https://github.com/tseemann/mlst, accessed 1 March 2022) and SpaTyper tools (https://github.com/mjsull/spa_typing, accessed 1 March 2022). Any genome with an unassigned MLST profile was uploaded to PubMLST (17) for curation (Table S3). To ensure a representative New Zealand bovine-derived *S. aureus* ST1 population, one ST1/CC1 isolate per farm per *spa* type was retained, yielding 196 bovine-derived *S. aureus* ST1 isolates for analysis, from 15 dairy regions (Fig. S2). Only human ST1/CC1 isolates were retained for analysis, resulting in a final data set of 205 ST1/CC1 isolates (196 bovine derived and nine human derived) used for further analysis.

### Hybrid genome assembly

A representative bovine-derived ST1 isolate (23EV612) underwent long-read MinION sequencing (R9.4.1 flow cell chemistry, Oxford Nanopore Technologies). Complete methodological details are provided in the Supplemental Material but are summarized below. Raw reads were base called with Guppy v.6.4.6 (26), filtered with Filtlong v.0.2 (https://github.com/rrwick/Filtlong, accessed on 1 March 2022), adapter-trimmed with Porechop v.0.2.4 (https://github.com/rrwick/Porechop, accessed on 1 March 2022), *de novo* assembled with Flye v.2.9.1 (27), and subsequently polished with Medaka v.1.6.0 (https://github.com/nanoporetech/medaka, accessed on 18 March 2022). Paired-end Illumina reads from the same isolate were aligned to the draft assembly, which was further polished using Pilon v.1.2.4 (28). The final assembly was processed using Circlator v.1.5.5 (29) to circularize and refine the genome, producing a complete chromosomal sequence.

### Phylogenetic analysis of ST1 New Zealand isolates

The final data set contained 520 New Zealand *S. aureus* ST1 genomes (Table S4), including 205 *S*. *aureus* ST1 genomes sequenced in this study, six human *S. aureus* genomes from the New Zealand Institute for Public Health and Forensic Science [PHF Science] (BioProject: PRJNA1029301), 177 New Zealand human *S. aureus* ST1 (hST1) genome assemblies (30–34) and 132 bovine *S. aureus* ST1 (bST1) genome sequences (19) obtained from the public sequence repositories (https://www.ebi.ac.uk/ena/browser/, accessed 15 February 2024).

Analysis was performed using Nullarbor v.2.0, with reference genome 23EV612 (GenBank: CP160024), Kraken2 (25) identification confidence set to 50% (0.5), and an updated *S. aureus* PubMLST (17) database for MLST typing. Resistome and virulome identification parameters were adjusted to query coverage of 55% and query identity of 90%. Query coverage was increased to detect repetitive or deleted genes, while query identity was increased to 90% to ensure accuracy. Resistome annotation utilized the Comprehensive Antibiotic Resistance Database (CARD) v.3.2.9 (35), and virulome identification was based on the Virulence Factor Full Database (VFDB) (accessed 15 March 2024) (36). Recombinant regions were detected and removed from the Snippy core alignment using Gubbins v.3.2.2 (37), using RapidNJ (38) as the tree-building algorithm and Jukes-Cantor (39) as the initial model. Phylogenetic trees were inferred with IQ-TREE v.2.26 (40) and 1,000 bootstrap replicates (41) and visualized using TreeViewer v.2.20 (42).

Heatmaps depicting virulence gene presence (Fig. S3 to S11) were generated in RStudio v.2024.04.2 with pheatmap package (43). A custom Python script (virulencegroup.py) was used to group isolates by host and virulence gene presence. These groupings were used to create the virulence heatmaps using the R script (Virulence_Heatmaps.r) (Fig. S3 to S11). To assess host clustering, the SNP-distance matrix (Table S5) was used to calculate SNP distances between isolates and to determine the inter- and intra-SNP averages using a custom Python script (InterIntraHost.py).

MGEs were identified via a custom ABRicate database containing 441 plasmid/phage sequences sourced from National Center for Biotechnology Information (https://www.ncbi.nlm.nih.gov/genome, accessed 5 December 2023), PAI DB v.2.0 (44), and relevant literature (45–47) (Table S6). Resistome/virulence genes on MGEs were annotated using Prokka v.1.45 (48), CARD v.3.2.9 (35), and VFDB (36), applying thresholds of 90% query identity and 55% query coverage. Associations between resistance/virulence genes and MGEs with either bovine or human hosts were tested for significance using a custom Python script (StatisticallySignificantGenes.py), employing either the chi-square test or Fisher’s exact tests.

### Phage characterization

The φSabovST1 phage (from 23EV612) was characterized by extracting φSaov3 genome coordinates (PAI DB: NC_017337_P4) via ABRicate, extending 10 kb on both ends, and exporting the sequence using samtools faidx (49). Phage annotation analysis was performed using PHASTEST (50) and Phage Scope (https://phagescope.deepomics.org/, accessed 9 July 2024), with visualization in Proksee (51) (Fig. S15). Genomic comparisons with φSaov3 were performed using Prokka v.1.14.6 (48) (annotation), OrthoFinder v.2.5.2 (52) (orthologous gene clustering), and Clinker v.0.0.31 (53) (synteny visualization). A core SNP phylogeny contextualizing φSabovST1 among siphoviruses sourced from reference 54 (Table S7) was generated using Snippy v.4.6.0 (https://github.com/tseemann/snippy, accessed 10 July 2024), SNP-dists v.0.8.2 (https://github.com/tseemann/snp-dists, accessed 10 July 2024), and IQ-TREE v.2.26 with 1,000 bootstrap replicates (40, 41), using φPV83 (GenBank: NC_002486.1) as a reference, and visualized in TreeViewer (42). Additionally, an average nucleotide identity was conducted using FastANI v.1.33 (https://github.com/ParBLiSS/FastANI, accessed 15 July 2024), and results were displayed as a heatmap using RStudio v.2024.04.2. The presence of φSabovST1 in global ST1 isolates was assessed using 98 bovine ST1 genomes sourced from public repositories (https://www.ebi.ac.uk/ena/browser/, accessed 30 August 2024; https://pathogen.watch/, accessed 30 August 2024) and evaluated using the custom MGE database (Table S8).

### Molecular clock analysis

The temporal signal was initially assessed using TempEst v.1.5.3 (55), evaluating root-to-tip divergence to determine suitability for molecular clock modeling. The *S. aureus* ST1 phylogeny revealed two distinct clusters (Clades 1 and 2, Fig. S12). The clades were analyzed separately, with core-genome alignments generated in Snippy v.4.6.0, with Clade 1 aligned to the best-quality short-read assembly identified as a human clinical isolate H8195 (Sequence Read Archive: SRR29758955) and Clade 2 aligned to 23EV612 (the bovine hybrid genome, GenBank: CP160024). Recombinant regions were identified and removed using Gubbins v.3.2.2 (37) and variant positions extracted with SNP-sites v.0.8.2. Root-to-tip divergence was reassessed in TempEst for both clades (Fig. S13 and S14), and genomes that exhibited significant deviations in root-to-tip regression (56) were excluded from downstream analyses (Table S9). Bayesian time-calibrated phylogenies were inferred using BEAST2 v.2.7.7 (57, 58), testing three independent Markov-chain Monte Carlo (MCMC) runs, each generating 20,000 trees for each model test (Tables S10 and S11). Model selection favored a Bayesian skyline optimized relaxed uncorrelated clock model (59, 60) for Clade 1 and a strict clock model for Clade 2 (57). Final analyses ran three independent MCMC simulations of 100 million generations per clade to estimate the time to the most recent common ancestor (tMRCA). Maximum credibility trees were visualized in FigTree v.1.4.4 (https://tree.bio.ed.ac.uk/software/figtree/, accessed 05 November 2024) and TreeViewer v.2.20 (42), with 95% highest posterior density (HPD) intervals representing the most probable tMRCA range. Full methodological details, priors, and parameter settings are described in the Supplemental Methods.

### Phenotypic expression of detected antimicrobial resistance genes

The Kirby-Bauer disk diffusion susceptibility test was conducted following the Vet01, 5th Edition, Clinical and Laboratory Standards Institute (CLSI) guidelines (61, 62). *S. aureus* strain American Type Culture Collection (ATCC 25923) was used as a reference control to verify QC ranges for each antibiotic. All *S. aureus* isolates were subcultured onto esculin sheep blood agar to confirm colony morphology before inoculum preparation. A 0.5 McFarland standard (Thermo Fisher) was used to standardize the bacterial density before inoculation. Phenotypic testing was conducted exclusively on the 205 ST1 isolates sequenced in this study (196 bovine and nine human) for susceptibility to penicillin and tetracycline (penicillin [10 IU] and tetracycline [30 µg], BBL Sensi-Discs antibiotic disks) on Mueller-Hinton agar.

Minimum inhibitory concentrations (MICs) were determined for 10 antimicrobial compounds against 24 bST1 *S. aureus* isolates, with *S. aureus* strain ATCC 25923 included as a QC strain using the Sensititre Mastitis CMV1AMAF Vet AST Plate (Thermo Fisher) in cation-adjusted Mueller-Hinton broth. The Vet-AST plate includes the following compounds: ampicillin, penicillin, erythromycin, oxacillin (supplemented with +2% NaCl), pirlimycin, penicillin/novobiocin, tetracycline, cephalothin, ceftiofur, and sulfadimethoxine. The MICs were interpreted according to CLSI susceptibility breakpoints (61).

## RESULTS

### Human and bovine *S. aureus* isolate collection

We analyzed *S. aureus* isolates collected from human and bovine sources across New Zealand to explore genetic differences between these populations. Bovine *S. aureus* isolates were sourced from bulk tank, aseptic quarter, and composite milk samples (Table S1; Fig. S1), while clinical human MSSA isolates were obtained from Pathlab (Table S2). We focused on ST1/CC1 strains; MLST typing was performed on all isolates. Fifty-four isolates had unassigned MLST types, and following submission to PubMLST for curation, 27 new sequence types were identified, including 14 within CC1, New Zealand’s dominant clonal complex (Table S3).

Bovine *S. aureus* isolates were collected from fifteen of New Zealand’s seventeen dairy farming regions (Fig. S2). Among these samples, 70% of sequenced bovine isolates belonged to ST1/CC1, and to prevent overrepresentation, only one ST1/CC1 isolate per farm with a unique *spa* type was retained, yielding 196 bST1 *S. aureus* and nine hST1 *S. aureus* isolates. Publicly available sequences expanded the data set to 520 *S*. *aureus* ST1 genomes: 328 bovine and 192 human (Table S4).

### Genomic assembly and characterization of the first bovine ST1 reference genome

Since no New Zealand ST1 reference genome existed, a hybrid genome assembly approach was employed to generate 23EV612, the first bST1 *S. aureus* reference genome. We used nanopore sequencing (MinION R.9.4.1) generating 1.4 million reads, and after filtering out poor-quality reads and barcodes/adaptors sequences, 1.2 million high-quality reads were retained with a mean Phred score of 17.8 (Table 1). High-quality Illumina reads were used to polish the nanopore assembly, resulting in a hybrid (Nanopore + Illumina) assembly of 23EV612 consisting of a single circular chromosome of 2,792,123 bp with a GC content of 32.89%.

**TABLE 1:** Sequencing and genomic characteristics of *Staphylococcus aureus* 23EV612

Characteristic	Result
Metadata	
Collection date	11 March 2020
Source	Bovine
Sample type	Aseptic Quarter 706 LB
Location	Te Kuiti, Western Uplands
Nanopore raw data	
Number of reads	1,434,650
Number of corrected reads	1,262,085
Read length N50	2,555,369
Phred mean read quality	17.8
Illumina raw data	
Total reads	4,001,993
Read length N50	127,115
Sequence quality (Phred score)	36
Hybrid assembly	
Total length	2,792,123
Read length N50	2,792,123
Number of contigs	1
GC content (%)	32.89
Contamination (%)	0.08
Completeness (%)	99.67
Genetic characteristics	
MLST	ST1
*spa* type	t114
GenBank accession number	CP160024
Antimicrobial resistance genes: CARD	*arlR*, *arlS*, *kdpD*, *lmrS*, *mepA*, *mepR*, *mgrA*, *norA*, *norC*, *sdrM*, *sepA*, and *tet*(38)
Virulence gene VFDB categoies	
Adherence	*aaa*, *atl*, *cna*, *clfA*, *clfB*, *eap/map*, *ebp*, *efb*, *emp*, *fnbA*, *fnbB*, *sasA*, *sasC*, *sdrC*, and *sdrD*
Enterotoxin	*seh*
Exoenzyme	*aur*, *coa*, *hysA*, *nuc*, *splA*, *splB*, *splC*, *splE*, *splF*, *sspA*, *sspB*, *sspC*, and *vWbp*
Exotoxin	*lukD*, *lukE*, *lukF-like*, *lukG*, *lukH*, *set19*, *set21*, *set26*, *set31*, *set32*, *set34*, *set36*, *set37*, *set39*, and *spa*
Hemolysin	*hlgA*, *hlgB*, *hlgC*, *hlb*, *hld*, and *hly*/*hla*
Immune modulation	*adsA*, *cap8A*, *cap8B*, *cap8C*, *cap8D*, *cap8E*, *cap8F*, *cap8G*, *cap8H*, *cap8I*, *cap8J*, *cap8K*, *cap8L*, *cap8M*, *cap8N*, *cap8O*, *cap8P*, *ebh*, and *sbi*
Intracellular/biofilm	*icaA*, *icaB*, *icaC*, *icaD*, and *icaR*
Others	*geh*, *harA*, *isdA*, *isdB*, *isdC*, *isdD*, *isdE*, *isdF*, *isdG*, *isdI*, *lip*, *sbnA*, *sbnB*, *sbnC*, *sbnD*, *sbnE*, *sbnF*, *sbnG*, *sbnH*, *sbnI*, *sfA*, *sfaB*, *sfaC*, *sfaD*, *sirA*, *sirB*, *sirC*, and *srtB*
VII secretion system	*esaA, esaB, esaD, esaE, esaG, essA, essB, essC, esxA, esxB, esxC*, and *esxD*

Using the CARD database, 12 efflux and regulatory genes were annotated in the 23EV612 genome (Table 1). These included multidrug efflux pumps such as *lmrS*, *sdrM*, *sepA*, and *mepA*, which are associated with antimicrobial resistance (AMR) (63). The *norA* and *norC* efflux pumps were identified, known to confer resistance to fluoroquinolones (63), while *tet*(38) is associated with tetracycline resistance (64, 65). Regulatory genes identified included *mepR*, *mgrA*, *arlS*, and *arlR* (66–68). Additionally, the *kdpD* gene, involved in the potassium transport system and implicated in antibiotic efflux, was detected (63).

The 23EV612 genome harbored 115 virulence-associated genes categorized into nine functional categories (Table 1). Among these, the *seh* gene, which encodes an enterotoxin, and the *ica* gene locus (*icaA-D*), associated with biofilm formation, were identified (69). Genes associated with capsular polysaccharide type 8 production linked to immune evasion and virulence were also present (70). Furthermore, the leukocidins *lukF-like* and *lukM*, which together form a two-component toxin known for its cytotoxic effects on bovine neutrophils, were annotated (71).

### Nine bST1 genomes clustering with hST1 genomes indicating potential spillover

**Fig 1:**
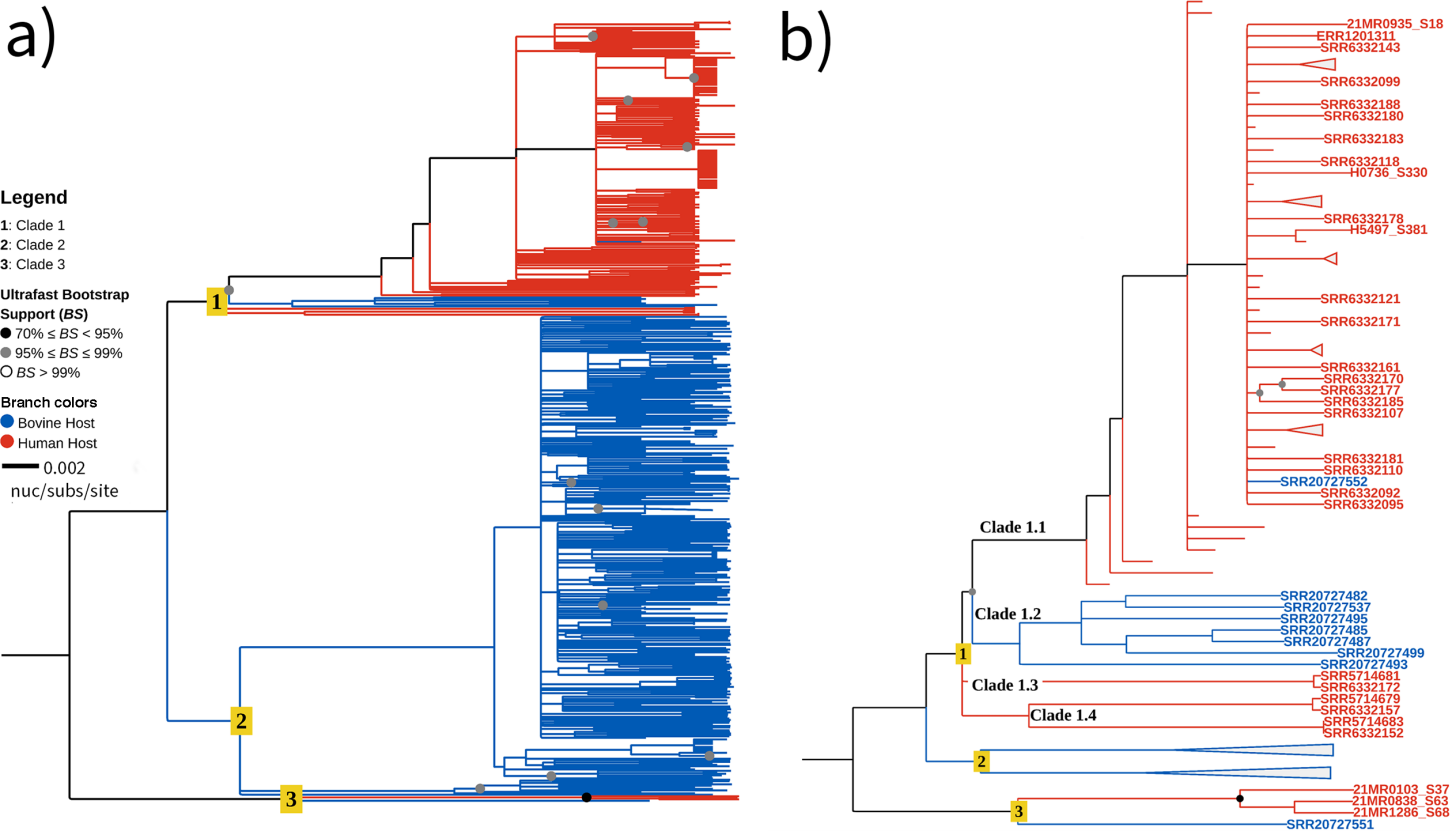
Maximum-likelihood phylogeny of 520 human and bovine *S. aureus* ST1 genomes from New Zealand. (**a**) Phylogeny generated from 19,953 SNPs using Snippy v.4.6.0 (https://github.com/tseemann/snippy), with 23EV612 as the reference genome. The tree was rooted using the outgroup (Clade 3) with visualization in TreeViewer (42). Branch colors indicate host origin: blue for bovine and red for human. Ancestral branches are colored to reflect the host origin if all descendant tips within that branch belong to the same host (e.g., Clade 2). Bootstrap support values are represented by colored dots: black (70%–95%) and gray (95%–99%). Clade numbers are shown on the nodes. The scale bar represents 0.002 substitutions per site. (**b**) Subsampled view of Clades 1 and 3 from panel A, highlighting intraclade diversity. Nodes and clades unrelated to focal groups are collapsed for clarity. Nine bovine *S. aureus* genomes in Clades 1.1, 1.2, and 3 clustered with human-associated genomes.

Phylogenetic analysis was performed using 23EV612 as the reference, generating a core-SNP phylogeny with 19,953 SNPs (Fig. 1a). The New Zealand ST1 phylogeny revealed three distinct clades, each supported by ≥99% bootstrap values (Fig. 1a). Clade 1 predominantly consists of hST1 genomes; however, several bST1 genomes are also present, signified by the blue branches in the tree (Fig. 1). Clade 2 consists exclusively of bST1 genomes, while Clade 3 contains a mix of both hST1 and bST1 genomes.

Clustering of bST1 with hST1 genomes was supported by ≥99% bootstrap values (Fig. 1b). In Clade 1.1, bST1 isolate SRR20727552 clusters with hST1 genomes, exhibiting an average pairwise SNP distance of 61 when compared to SRR6332092, SRR6332095, and ERR1201315 (Table S12A). Clade 1.2 contains seven bST1 genomes sharing a recent ancestor with Clades 1.3 and 1.4, with an average SNP difference of 319 (Table S12B; Fig. 1b). In Clade 3, the bST1 genome SRR20727551 is closely related to three hST1 genomes, separated by an average of 273 SNPs (Table S12C; Fig. 1b).

SNP differences between bovine-bovine (intrahost), human-human (intrahost), and bovine-human (interhost) genome pairs showed average intrahost distances of 127.70 (bovine) and 130.79 (human) (Mann-Whitney *P* value < 0.0001), while interhost SNPs averaged 229.61. Clades 1.2 and 3 both exhibited higher than average interhost SNP distances (319 and 273 SNPs, respectively), while Clade 1 showed a markedly lower interhost SNP distance of 61 SNPs, below the overall interhost average (Table S5).

### Resistome analysis highlights eight significant genes in hST1

AMR genes in human and bovine *S. aureus* genomes were assessed using the CARD database, excluding singleton genes (found in only one isolate). We identified 22 AMR genes across the 520 ST1 genomes (Table S13). Twelve AMR genes, mainly efflux pumps and regulators, were present in all isolates (Fisher’s exact *P* value > 0.05) (Table 2). The quaternary ammonium compound resistance gene (*qacB*) was found only in seven bST1 genomes, but Fisher’s exact test yielded a *P* value of >0.05, indicating no statistically significant association (Table 2). The penicillin resistance gene (*blaZ*) was the most prevalent AMR gene in bST1 genomes (12%). Five genes (*blaZ*, *qacA*, *fusC*, *ermC*, and *mupA*) were significantly enriched in hST1 (*P* value < 0.01) (Table 2).

**TABLE 2:** Statistical analysis comparing the presence or absence of AMR genes of bST1 and hST1 genomes^*c*^

AMR gene^*a*^	Presence in bST1 genomes	Presence in hST1 genomes	*P* value and statistical test used^*b*^	Odds ratio^*b*^	95% confidence interval^*b*^	Host association
*ant(4′)-la*	0/328 (0%)	12/192 (6%)	<0.0001 (FE)	0.022	0.0013–0.37	Human
*blaZ*	38/328 (12%)	164/192 (85%)	<0.0001 (CS)	0.022	0.013–0.038	Human
*dfrG*	0/328 (0%)	6/192 (3%)	<0.01 (FE)	0.044	0.0020-0.78	Human
*ermC*	2/328 (0.6%)	10/192 (5%)	<0.01 (FE)	0.11	0.024–0.52	Human
*fusC*	1/328 (0.3%)	168/192 (88%)	<0.0001 (CS)	0.00040	0.000059–0.0030	Human
*mecA* and *mecR1*	0/328 (0%)	33/192 (17%)	<0.0001 (CS)	0.0070	0.00044–0.12	Human
*mepA*, *mepR*, *mgrA*, *sdrM*, *tet*(38)*, arlS*, *arlR*, *lmrS*, *norA*, *norC*, *kdpD*, and *sepA*	328/328 (100%)	192/192 (100%)	1.00 (FE)	1.7	0.034–86.0	No host significance, genes present in all genomes
*mupA*	1/328 (0.3%)	121/192 (63%)	<0.0001 (CS)	0.0020	0.00025–0.013	Human
*qacA*	12/328 (4%)	110/192 (57%)	<0.0001 (CS)	0.032	0.015–0.054	Human
*qacB*	7/328 (2%)	0/192 (0%)	0.11 (FE)	8.9	0.51–158	No host significance

^*a*^ AMR genes were identified using ABRicate with the CARD database.

^*b*^ The *P* value, odds ratio, and the 95% CI were computed using a custom Python script (StatisticallySignificantGenes.py). The CS test was chosen for statistical analysis; however, if the expected frequency was ≤5, the FE test was used. All *P *values were corrected using Benjamini-Hochberg (false discovery rate) method. Genes with *P* ≥ 0.05 were considered not significantly associated with either host.

^*c*^ AMR, antimicrobial resistance; bST1, bovine *S. aureus* ST1; CI, confidence interval; CS, chi-square; FE, Fisher’s exact; hST1, human *S. aureus* ST1.

The methicillin resistance gene (*mecA*) and regulator (*mecR1*), dihydrofolate reductase (*dfrG*), and aminoglycoside resistance gene [ant(*4′)-la*] were absent in bST1 genomes but were significantly associated with hST1 genomes (*P* value < 0.01) (Table 2). *mecR1* query coverage in 33 hST1 genomes was 55.46%, and *fusC* query coverage in 7 hST1 genomes (56%–79.8%) was below the standard ABRicate query coverage threshold of 80% (Table S13). One bST1 and 24 hST1 showed a reduced *blaZ* query coverage of 67.7% (Table S13).

Phenotypic antimicrobial testing was conducted exclusively on the 205 sequenced isolates (196 bST1 and nine hST1) to validate our resistome findings (Table 3). While the full data set contained 520 isolates, the remaining 315 were publicly available sequences from repositories, preventing direct phenotypic validation due to the lack of physical access to these isolates. Testing focused on isolates harboring *blaZ* and *tet*(38), as these genes were the most commonly identified resistance genes across both host groups, and well-established testing protocols were available. Several other resistance genes, such as *fusC* (fusidic acid), *ermC* (macrolides/lincosamides resistance), *mecA* (β-lactam/methicillin), *qacA/qacB* (biocide resistance), and *mupA* (mupirocin resistance), were predominantly identified in publicly available genomes. However, the lack of access to these isolates, combined with the absence of standardized testing protocols, particularly for *qacA/qacB*, made resistome validation for these genes unfeasible (Table 3; Table S14).

**TABLE 3:** Genotypic vs phenotypic validation of *blaZ* and *tet*(38) in 205 sequenced *S. aureus* ST1 isolates^*e*^^,^^*f*^

Host-AMR group	AMR gene query coverage (%)^*a*^	Total isolates in Host-AMR group ^*a*^	Isolates phenotypically tested by disk diffusion^*b*^	Isolates phenotypically tested by MIC^*c*^	Genotype vs phenotype concordance^*d*^
hST1 *blaZ*+	80–100	140	5	0	100% resistant
	67.7	24	1	0	100% resistant
bST1 *blaZ*+	80–100	37	22	19	100% resistant
	67.7	1	1	1	100% resistant
hST1 *blaZ*−	–^*g*^	28	3	0	100% susceptible
bST1 *blaZ*−	–^*g*^	290	173	4	100% susceptible
hST1 *tet*(38)	80–100	192	9	0	100% susceptible
bST1 *tet*(38)	80–100	328	196	24	100% susceptible

^*a*^ AMR gene detection and query coverage was determined by using the CARD database in ABRicate using the full data set of 520 *S*. *aureus* ST1 isolates.

^*b*^ The Kirby-Bauer disk diffusion susceptibility test was conducted using Vet01, 5th Edition, Clinical and Laboratory Standards Institute (CLSI) guidelines and interpretations of breakpoints. Disk diffusion was limited to the 196 bST1 and 9 hST1 isolates due to lack of physical access to isolates corresponding to publicly available genomes.

^*c*^ MIC testing was only completed on 24 bST1 isolates.

^*d*^ Concordance (%) indicates agreement between genotype (ABRicate detection) and phenotype (disk diffusion and/or broth dilution).

^*e*^ The table presents AMR gene presence or absence across human (hST1) and bovine (bST1). Phenotypic validation was conducted through disk diffusion (205 sequenced isolates) and MIC testing on a randomly selected subset of 24 bST1 isolates. Genotype-phenotype validation indicates whether resistance or susceptibility aligns with the genetic findings.

^*f*^ Zone diameters and MIC results are provided in Table S14.

^*g*^ “–” indicates not applicable.

Disk diffusion confirmed phenotypic resistance in *blaZ-*positive isolates, including two with 67.7% query coverage (Table 3; Table S14). In contrast, *tet*(38), encoding a tetracycline-specific efflux pump, did not correlate with phenotypic resistance. Broth microdilution MIC testing was conducted on a randomly selected subset of 24 bST1 *S. aureus* isolates from the 196 sequenced bST1 *S. aureus* genomes. All tested isolates were susceptible to erythromycin, oxacillin, pirlimycin, penicillin/novobiocin, tetracycline, cephalothin, ceftiofur, and sulfadimethoxine. However, 20 of the 24 isolates exhibited resistance to penicillin (MIC > 0.25 µg/mL), and 16 were resistant to ampicillin (MIC > 1 µg/mL) (Table S14). Notably, bST1 genomes from Clades 1.1, 1.2, and 3 were of particular interest for their AMR profiles (Fig. 1b). The *blaZ* gene was detected in two bST1 genomes: SRR20727482 in Clade 1.2 and SRR20727551 in Clade 3. Additionally, SRR20727552 in Clade 1.1 harbored *blaZ*, *fusC*, *qacA*, and *mupA*, making it the only bST1 genome to carry both *fusC* and *mupA*.

### Virulence gene analysis shows evidence for niche specialization

Virulome analysis using the VFDB database identified 132 virulence genes among the 520 ST1 *S. aureus* genomes. After excluding singleton genes and 3 genes with undefined function, 124 unique virulence genes were retained (Table S15A and B). Allelic variants of *clfA* (clumping factor A), *hlb* (β-hemolysin), *esaG* (Type VII secretion system), and *splE* (serine protease E) were included if they lacked premature stop codons (Table S15A).

Eighty virulence genes were present in all bST1 and hST1 genomes, forming a putative core virulome associated with general *S. aureus* survival and pathogenicity (Table S15B). These genes were primarily involved in adhesion (Fig. S3), exoenzyme activity (Fig. S5), exotoxin production (Fig. S6), immune modulation (Fig. S8), biofilm formation (Fig. S9), secretion systems (Fig. S10), and other virulence-related functions (Fig. S11).

Eighteen genes showed statistically significant differences in prevalence between hST1 and bST1 genomes (*P* < 0.05), suggesting host-specific associated variation (Table 4; Table S16). Notably, *hlb*(1) was predominantly associated with bST1 genomes, while *hlb*(2) was more prevalent in hST1 genomes (Table 4; Fig. S7). SRR20727552, in Clade 1.1, was the only bST1 genome harboring *hlb*(2). The serine protease gene *splE*(1) was statistically associated with bST1 genomes (chi-square *P* value < 0.0001) (Table 4; Fig. S5).

**TABLE 4:** Statistically significant virulence genes identified by the presence and absence analysis in bST1 and hST1 genomes^*c*^

Virulence gene^*a*^	Presence in bST1 genomes	Presence in hST1 genomes	*P* value and statistical test used^*b*^	Odds ratio^*b*^	95% confidence interval^*b*^	Host association
β-Hemolysin: *hlb(1*)	327/328 (99%)	8/192 (4%)	<0.0001 (CS)	7,500	930–61,000	Bovine
β-Hemolysin: *hlb(2)*	1/328 (0.3%)	184/192 (93%)	<0.0001 (CS)	0.00013	0.000017–0.0011	Human
Capsule: *cap8H*	323/328 (98%)	176/192 (92%)	<0.001 (CS)	5.8	2.1–16.0	Bovine
Chemotaxis-inhibiting protein CHIPS: *chp*	0/328 (0%)	83/192 (43%)	<0.0001 (CS)	0.0020	0.00012–0.033	Human
Collagen adhesion precursor: *cna*	129/328 (39%)	140/192 (73%)	<0.0001 (CS)	0.25	0.16–0.36	Human
Enterotoxin type A: *sea*	1/328 (0.3%)	178/192 (93%)	<0.0001 (CS)	0.00024	0.000032–0.0018	Human
Enterotoxin type B: *seb*	0/328 (0%)	5/192 (3%)	<0.05 (FE)	0.052	0.0029–0.94	Human
Enterotoxin type K: *selk*	1/328 (0.3%)	94/192 (49%)	<0.0001 (CS)	0.0032	0.00044–0.23	Human
Enterotoxin type Q: *selq*	1/328 (0.3%)	98/192 (51%)	<0.0001 (CS)	0.0029	0.00041–0.021	Human
Leukocidin: *lukF-like*	278/328 (85%)	0/192 (0%)	<0.0001 (CS)	2100	130–35,000	Bovine
Leukocidin: *lukF-PV*	1/328 (0.3%)	140/192 (73%)	<0.0001 (CS)	0.0011	0.00016–0.0083	Human
Leukocidin: *lukM*	278/328 (85%)	0/192 (0%)	<0.0001 (CS)	2100	130–35,000	Bovine
Leukocidin: *lukS-PV*	1/328 (0.3%)	140/192 (73%)	<0.0001 (CS)	0.0011	0.00016–0.0083	Human
Serine protease E: *splE*(1)	318/328 (97%)	15/192 (8%)	<0.0001 (CS)	380	170–850	Bovine
Staphylococcal complement inhibitor: *scn*	1/328 (0.3%)	187/192 (97%)	<0.0001 (CS)	0.000082	0.0000095–0.00072	Human
Staphylokinase: *sak*	1/328 (0.3%)	187/192 (87%)	<0.0001 (CS)	0.000082	0.0000095–0.00072	Human
Type VII secretion system: *esaG*(1)	328/328 (100%)	185/192 (96%)	<0.01 (FE)	27	1.5–470.0	Bovine
Type VII secretion system: *esaG*(2)	0/328 (0%)	7/192 (4%)	<0.01 (FE)	0.038	0.0021–0.66	Human

^*a*^ Virulence genes were identified using ABRicate with the VFDB database.

^*b*^ The* P *value, odds ratio, and the 95% CI were computed using a custom Python script (StatisticallySignificantGenes.py). The CS test was chosen for statistical analysis; however, if the expected frequency was ≤5, the FE test was used. All* P* values were corrected using the Benjamini-Hochberg (false discovery rate) method.

^*c*^ CS, chi-square; FE, Fisher’s exact.

Enterotoxin genes (*sea, seb, selk*, and *selq*) were predominantly detected in hST1 genomes, except SRR20727552 was the only bST1 genome carrying multiple enterotoxins (*sea*, *selk*, and *selq*) (Fig. S4). Immune evasion genes (*sak*, *scn*, and *chp*) were significantly more associated with hST1 genomes (*P* value < 0.01), while SRR20727552 was the only bST1 genome with *sak* and *scn* (Fig. S5 and S8). Leukocidins showed host-specific distribution; human-adapted leukocidins (*lukF-PV*/*lukS-PV*) were detected in 72.9% of hST1 genomes (*P* value < 0.01), whereas bovine-adapted leukocidins (*lukF-like*/*lukM*) were present in 82.2% of bST1 genomes (*P* value < 0.01) (Fig. S6). SRR20727552 was again the only bST1 genome carrying the human leukocidins, and no bST1 genomes in Clade 1.2 or 3 carried either leukocidin type. Of the 18 statistically significant genes, six were more prevalent in bST1 and 12 in hST1 genomes (Table 4).

### Discovery of φSabovST1, a prophage exclusively present in bST1 genomes

To examine MGE distribution in hST1 and bST1 genomes, an MGE database of 441 *S. aureus* plasmids, pathogenicity islands, and phages was constructed (Tables S6 and S17). We annotated 25 MGEs carrying AMR and virulence genes, including seven plasmids harboring the *blaZ* gene and three φSa3 temperate phages. The φSa3 phages integrate into the *hlb* gene locus and carry human immune evasion cluster genes (*sak*, *scn*, and *chp*) (Table S18) (72). In these isolates, *hlb*, *sak*, and *scn* were all co-located on the same chromosome, consistent with phage integration at the *hlb* site. Notably, while φSa3 was predominantly associated with hST1 isolates, we also detected φSa3 (Pai DB: NC_003923_P6, GenBank: NC_003923) in one bovine isolate (SRR20727552).

**Fig 2:**
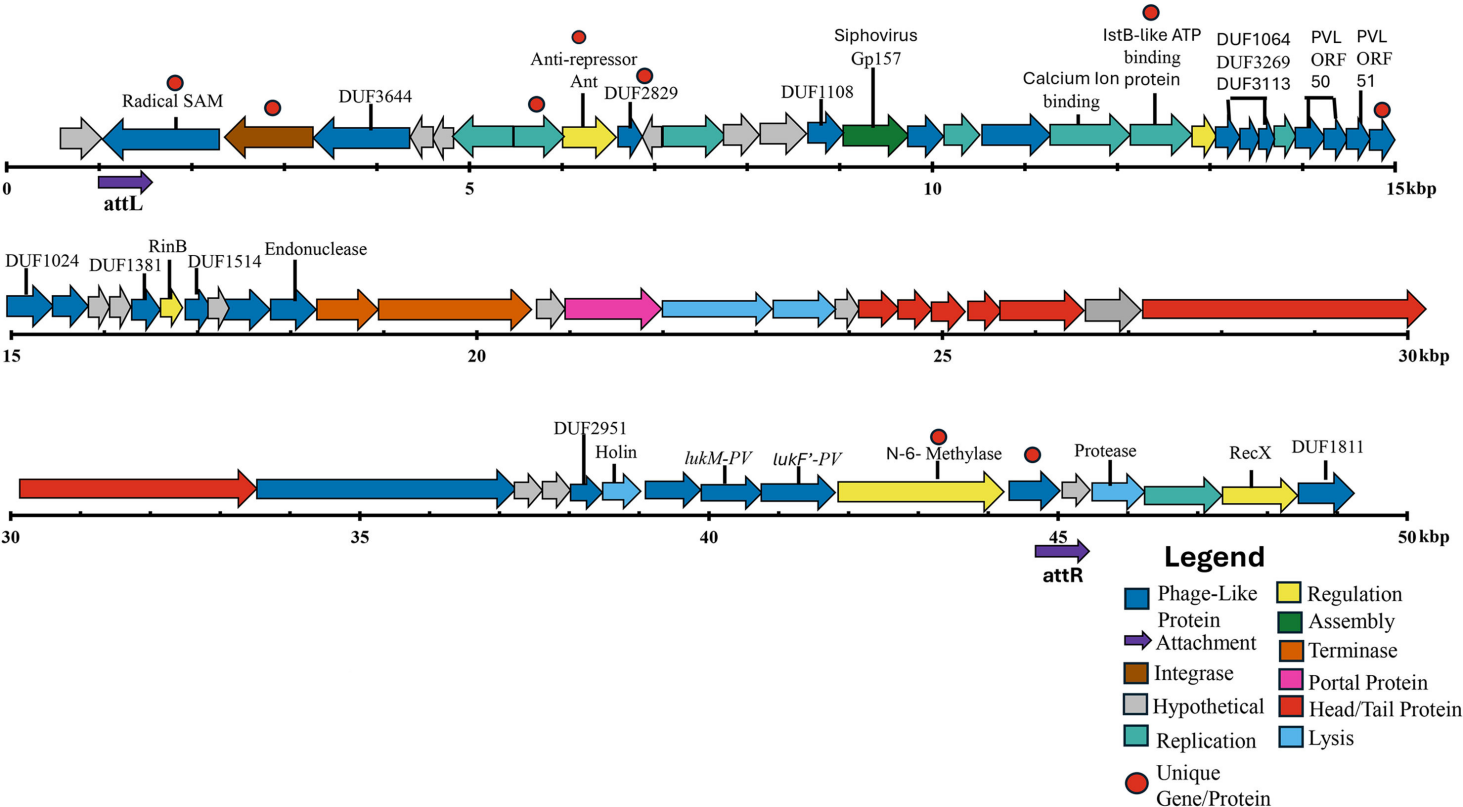
Schematic gene representation of φSabovST1 prophage, showing annotated structural and functional proteins. φSabovST1 is classified as a siphovirus based on its assembly machinery. The novel prophage encodes the bovine-adapted leukocidins: *lukF-like/lukM.* Each arrow represents an open reading frame (ORF) and its orientation. The φSabovST1 integrates into the host genome at the attL and attR attachment sites (marked by purple arrows), which are flanked by hypothetical proteins. Each ORF is color-coded to its functional annotation: dark blue, phage-like protein; brown, integrase; gray, hypothetical; teal, replication; yellow, regulation; dark green, assembly; orange, terminase; pink, portal protein; red, head/tail protein; and light blue, lysis. The nine red circles above specific ORFs indicate unique proteins to φSabovST1 as determined by OrthoFinder (52).

φSaov3 (Pai DB: NC_017337_P4, GenBank: NC_017337) (73), a phage with ovine origin, was significantly associated with bST1 genomes (false discovery rate [FDR] corrected, *P* value < 0.0001), detected in 275 of 328 bST1 genomes and entirely absent in hST1 genomes (Tables S17 and S19). ABRicate analysis revealed 98% identity and approximately 62% coverage, suggesting a high similarity to φSaov3 but partial match to the reference sequence. To further characterize this finding, we examined the ST1 reference genome 23EV612, where an intact 44,213 bp prophage was identified. This prophage closely resembled φPV83 (74) and carried the bovine-adapted leukocidin genes *lukF-like*/*lukM* (75, 76), similar to those found in φPV83 and φSaov3. The newly identified phage was designated φSabovST1 and classified as a siphovirus, with defined attL and attR attachment sites, integrated between two hypothetical proteins (Fig. 2; Fig. S15).

**Fig 3:**
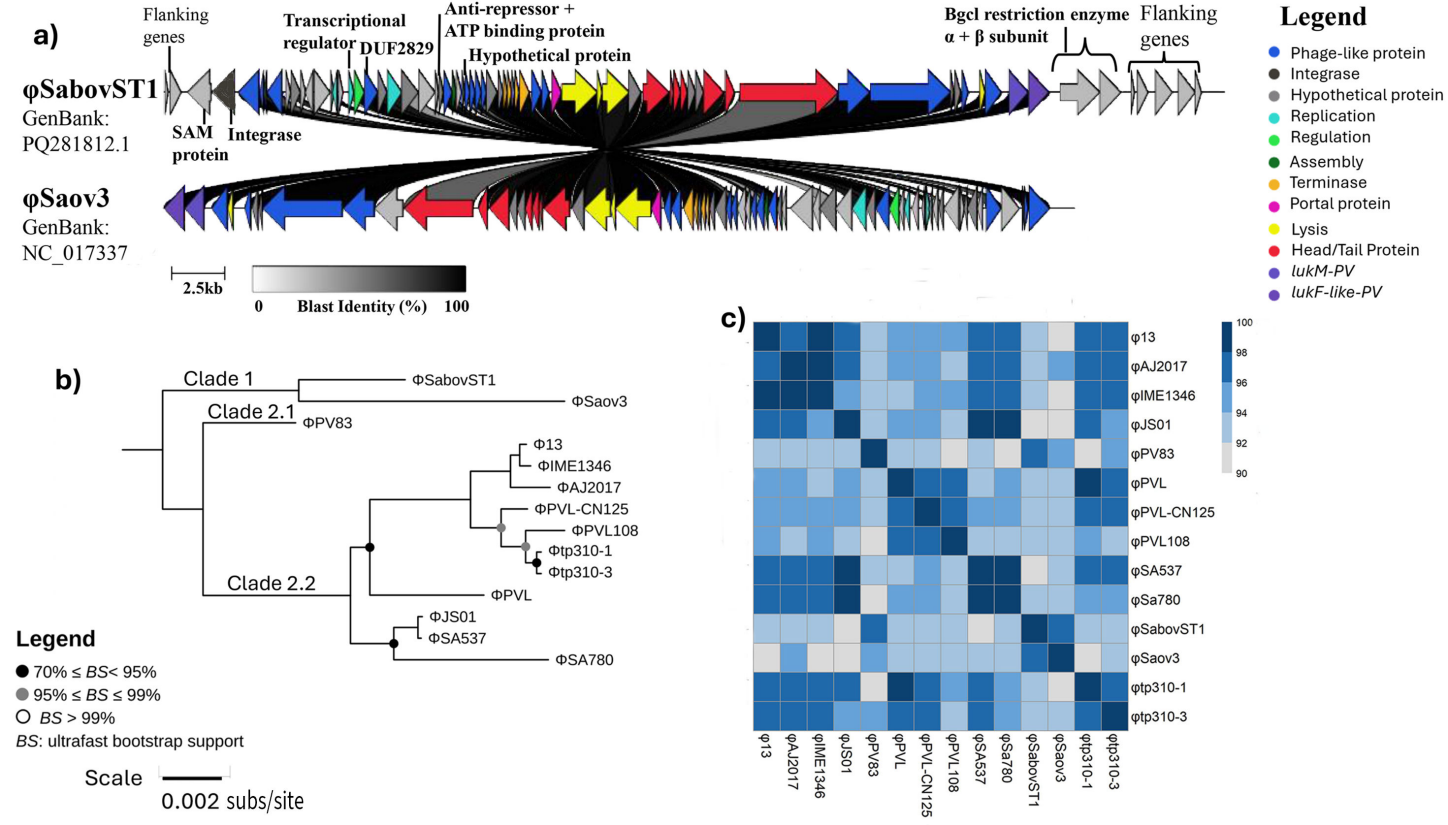
Comparative genomic analysis for φSabovST1 compared to related phages. (**a**) Gene synteny plot between φSabovST1 and φSaov3 visualized with Clinker (53). BLAST identity is shown from 0% (white), 50% (gray), to 100% (black). Arrows indicate open reading frames (ORFs) and their transcriptional orientation. Each ORF is color-coded by predicted function: phage-like, dark blue; integrase, gray; hypothetical, light gray; replication, light blue; regulation, light green; assembly, dark green; terminase, orange; portal protein, pink; lysis, yellow; head/tail, red; *lukM*, light purple; and *lukF′-PV*, dark purple. Unique φSabovST1 proteins identified by OrthoFinder (52) include a SAM protein, integrase, a transcriptional regulator, an antirepressor, an ATP-binding protein, a hypothetical protein, DUF2829 domain, and α/β subunits of the BgcI restriction enzyme. (**b**) Phylogenetic relationships inferred using maximum likelihood from 818 core SNPs across φPV83 (74) (reference genome), φSaov3 (73), φSabovST1, and cluster B7 siphoviruses (54). φSaov3 and φSabovST1 cluster together in Clade 1, while φPV83 forms Clade 2.1, and cluster B7 siphoviruses form Clade 2.2. Bootstrap support values are represented by colored dots: black dots (70%–95%) or gray (95%–99%). Scale bar = 0.002 substitutions/site. (**c**) Average nucleotide identity (ANI) heatmap generated by FastANI and visualized using pheatmap (43). The heatmap compares cluster B7 siphoviruses, including φPV83, φSaov3, and φSabovST1. ANI similarities were 94%–98% between φPV83 and φSabovST1, and 96%–98% between φSaov3 and φSabovST1.

Comparative analysis using OrthoFinder revealed that φSabovST1 and φSaov3 shared 52 orthologs, while 9 orthologs were unique to φSabovST1. These included genes encoding a SAM domain-containing protein, integrase, transcriptional regulator, antirepressor, BcgI restriction-modification enzyme subunits, and a hypothetical protein of unknown function (Fig. 2 and 3a). φSabovST1 was detected in 83.8% of bST1 genomes, was completely absent from hST1 genomes, and restricted to Clade 2, with no presence in Clades 1.1, 1.2, and 3.

To assess if φSabovST1 was unique to New Zealand ST1 isolates, we screened 98 publicly available bovine ST1 genomes using a custom MGE reference database and ABRicate. Initially, φSaov3 was detected in 19 genomes with high sequence identity (98% identity) but partial coverage (~60% coverage). Upon incorporating φSabovST1 into the MGE database, these same isolates were identified as carrying φSabovST1. Among them, seven genomes—five from Ireland and two from Malaysia—showed full-length matches with 100% query coverage and 99.99% sequence identity to φSabovST1 (Table S8).

To explore the evolutionary relationship among related phages, we constructed a phylogeny including cluster B7 siphoviruses defined by reference 54, along with φSaov3 and φSabovST1. Using φPV83 as the reference, a core SNP phylogeny revealed that φSaov3 and φSabovST1 clustered together in Clade 1, separated by 284 SNPs with ≥99% bootstrap support (Table S20; Fig. 3b). In contrast, φPV83 formed a distinct lineage in Clade 2.1, differing from φSabovST1 by 388 SNPs. Average nucleotide identity (ANI) similarities ranged from 94% to 98% between φPV83 and φSabovST1 and from 96% to 98% between φSaov3 and φSabovST1 (Fig. 3c). By evaluating the gene content, phylogeny clustering, and high ANI similarities between φSaov3 and φSabovST1, we hypothesize that φSabovST1 is a distinct strain closely related to φSaov3.

### Temporal analysis of Clade 2 indicates acquisition of φSabovST1 phage in the early 2000s

As a final assessment of the 520 ST1 isolates, we investigated the temporal origin of the lineage within New Zealand. Initial analysis using tip-dating methods (TempEst) revealed no reliable temporal signal (*R*^2^ = 0.001), indicating a poor correlation between genetic divergence and sampling date, and suggested the data set was not suitable for molecular clock analysis as a whole (Fig. S12).

However, the phylogeny showed two well-supported clusters: Clades 1 and 2 (Fig. 1). To refine resolution, genomes deviating from the TempEst regression line were removed. For Clade 1, 65 genomes from Clade 1.1 and additional genomes from Clades 1.2, 1.3, and 1.4 primarily sourced from public repositories were excluded (Table S9). The remaining 119 genomes exhibited a weak temporal signal (*R*^2^ = 0.11), likely due to uneven sampling, with most genomes collected in either 2014 or 2019 (Fig. S13). In Clade 2, we excluded 29 genomes that fit poorly with the regression model, leaving 292 genomes with a moderate temporal signal (*R*^2^ = 0.62) (Fig. S14). Based on these findings, we proceeded with molecular clock analyses for both clades using Bayesian ancestral state reconstruction (57, 58).

**Fig 4:**
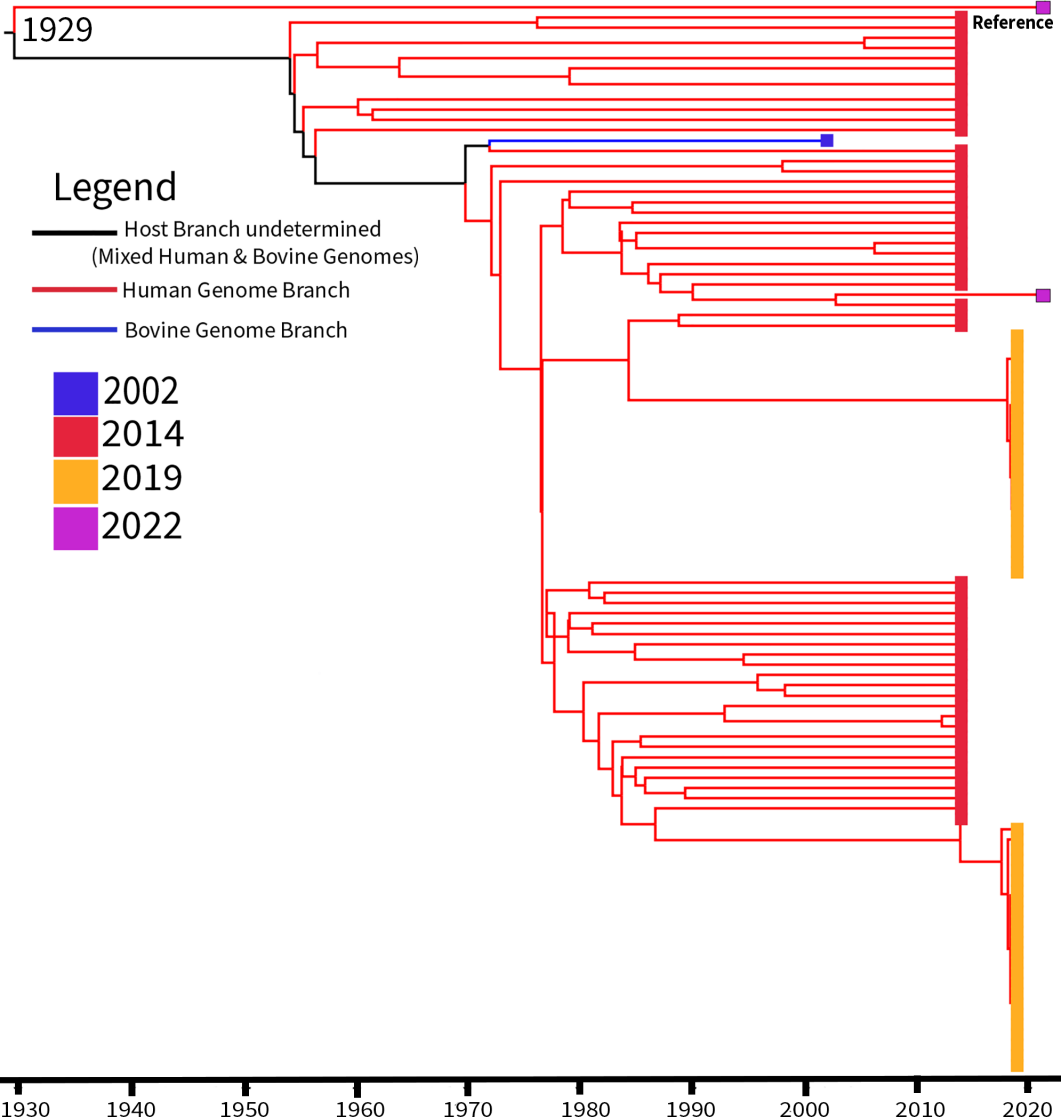
Time-scaled phylogenetic reconstruction of 119 *S. aureus* ST1 New Zealand genomes from Clade 1. The phylogeny is midpoint rooted and determined from 2,414 variant sites, with the short-read H8195 isolate (Sequence Read Archive: SRR29758955) as the reference strain (labeled as reference). The time to the most recent common ancestor was determined as 1929 (95% highest posterior density: 1765–1982) using the optimized relaxed uncorrelated clock model with the Bayesian skyline model in BEAST2 v.2.7.7 (57, 58). Branches are colored by host: blue (bovine) and red (human). Nodes are color-coded by the sampling year of collection: blue, 2002; red, 2014; yellow, 2019; and pink, 2022. The bovine isolate SRR20727552 (blue branch) and a closely related human isolate shared a common ancestor dated to 1971, suggesting host-transmission events in the last ~50 years. Notably, the majority of isolates were collected between 2014 and 2019.

For Clade 1, the nested sampling algorithm favored the optimized relaxed uncorrelated clock model (marginal likelihood: −18,526.30; standard deviation [SD], ±4.02) compared to the strict clock model (−18,537.36; SD, ±4.00). The tMRCA was estimated to be 1929 (95% HPD: 1765-1982) (Fig. 4), based on 2,414 variant sites with a genome-wide mutation rate of 7.37 × 10^−7^ substitutions per site per year. Interestingly, SRR20727552 and a closely related human isolate shared a common ancestor dated to 1971, suggesting potential host-transmission events within the last ~50 years. The weak temporal signal, the mutation rate, and limited variation may be attributable to the skewed sampling distribution.

**Fig 5:**
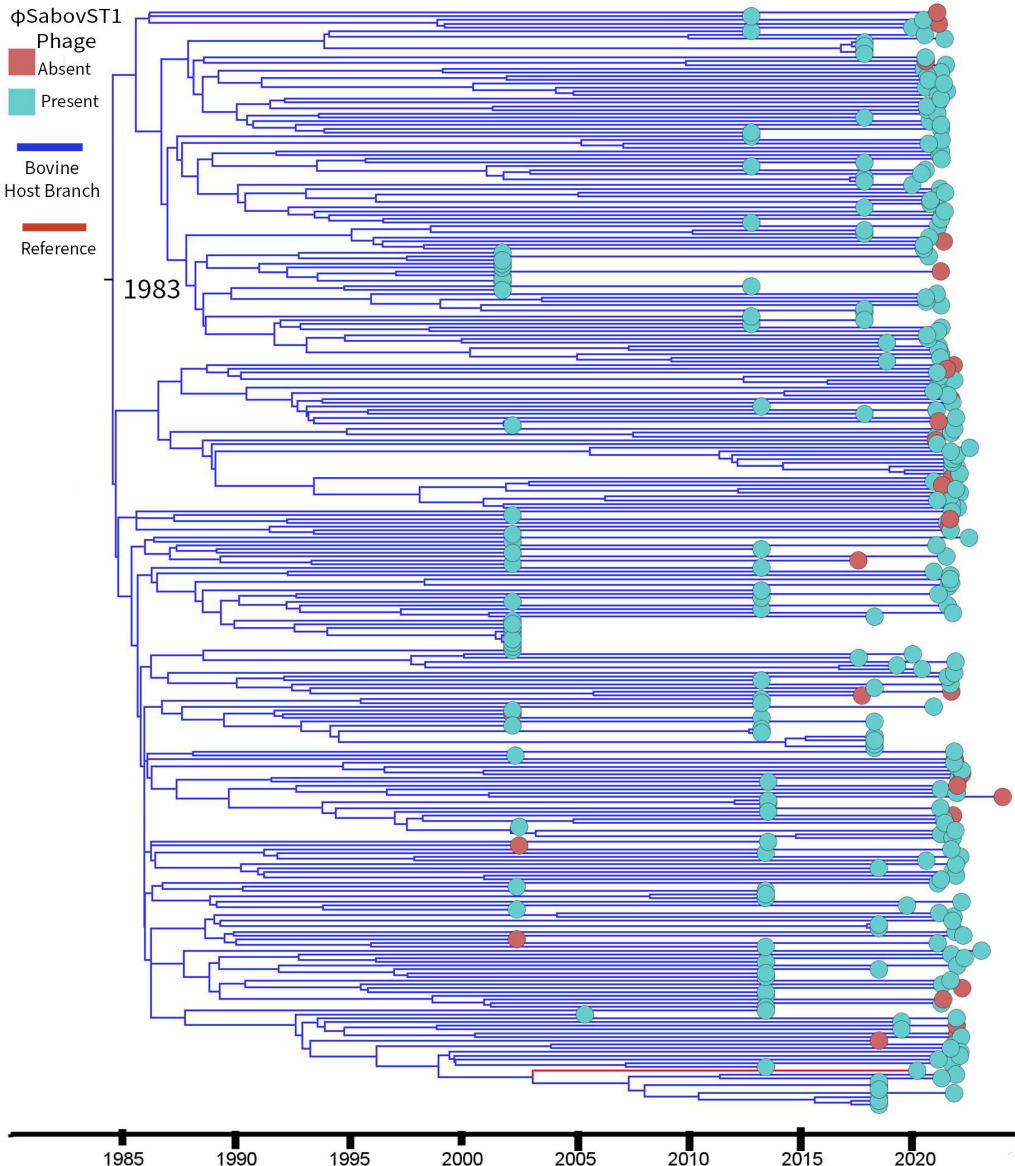
Time-scaled phylogeny of 292 Clade 2 *S. aureus* ST1 New Zealand isolates, highlighting the distribution of the φSabovST1 phage. The phylogeny is midpoint rooted and determined from 16,554 variant sites, with 23EV612 (GenBank: CP160024) as the reference strain (indicated by a red line at the midpoint). The time to the most recent common ancestor was determined as 1983 (95% highest posterior density: 1981–1986) using the strict clock with the Bayesian skyline model in BEAST2 v.2.7.7 (57, 58). Branches are colored by host: blue (bovine). Blue circles indicate the presence of φSabovST1, while red circles indicate its absence. Phage-positive isolates are interspersed throughout the phylogeny, while phage-negative isolates are not restricted to a single lineage. The patchy distribution suggests multiple independent events of phage acquisition or loss.

For Clade 2, nested sampling algorithm supported a strict clock model (marginal likelihood: −184,990.93; SD, ±4.45) compared to the optimized relaxed uncorrelated clock model (−186,817.47; SD, ±4.60). The tMRCA was estimated at 1983 (95% HPD: 1981–1986) (Fig. 5), based on 16,554 variant sites with a genome-wide mutation rate of 1.35 × 10^−6^ substitutions per site per year, aligning with prior estimates of *S. aureus* mutation rates in New Zealand (ST97: 1.32 × 10^−6^ substitutions per site per year) (77). Given that φSabovST1 was restricted to Clade 2, its presence was mapped across the phylogeny. First detected intermittently between 2000 and 2005, φSabovST1-negative isolates were dispersed across the tree rather than being confined to a single lineage, suggesting multiple independent acquisition or loss events rather than a single point of origin.

## DISCUSSION

This study presents one of the most comprehensive genomic characterizations of *S. aureus* ST1/CC1 from human and bovine sources in New Zealand. While previous research has demonstrated host specialization in ruminants (22), our integration of Illumina and Nanopore sequencing with population genomics and phylodynamic approaches reveals significant divergence in AMR, virulence, and MGE profiles between human and bovine-derived ST1 isolates. These findings highlight how the presence of the *S. aureus* ST1/CC1 lineage in both human and bovine populations in New Zealand challenges the assumption of strict host specificity, a concept also brought into question by the detection of the MRSA AK3 strain in both clinical and potential non-clinical reservoirs (78). This underscores the value of a One Health framework for understanding bacterial transmission, AMR, and pathogen ecology across human and animal populations, both locally and globally.

A key finding was the identification of a novel prophage, φSabovST1, restricted only to bST1 genomes within Clade 2, highly similar to the ovine-origin prophage φSaov3 (73), and encodes bovine-adaptive leukocidins (*lukF-like*/*lukM*), which may enhance pathogenicity in dairy cattle (71, 75, 76, 79, 80). Clade 2 emerged around 1983, with φSabovST1 first appearing in the early 2000s. Its patchy distribution suggests multiple acquisition or loss events rather than a single clonal expansion. Although φSabovST1 was present in 83.8% of bST1 *S. aureus* isolates in this study, the absence of *lukF-like/lukM* in asymptomatic bovine *S. aureus* genomes from another New Zealand study (81) points to a complex interplay between prophage carriage, host adaptation, and disease expression. Notably, φSabovST1 was also detected in five Irish genomes, indicating potential international relevance, though limited global ST1 data prevent broader conclusions. New Zealand’s geographical isolation and strict biosecurity have likely contributed to a distinct *S. aureus* population structure, characterized by limited gene flow compared to other regions (15, 82). Despite increasing global connectivity, New Zealand is a rare and valuable case study and highlights both the benefits and challenges of sustained isolation. While this preserves local genomic evolution, it may heighten susceptibility to novel pathogen incursions (15, 83–85). New Zealand’s microbial distinctiveness provides insights into AMR boundaries and evolutionary dynamics under geographical isolation, offering insights increasingly relevant to global One Health strategies.

We detected the φSa3 prophage, which carries human-specific immune evasion genes (*sak*, *scn*, and *chp*), in human isolates and one bovine genome (SRR20727552). φSa3 typically integrates into the *hlb* locus, potentially disrupting β-hemolysin function (8, 86, 87). While *hlb*, *sak*, and *scn* were co-located in several genomes, the functional impact of φSa3 integration remains uncertain, as β-hemolysin activity has been observed in some cases despite the presence of φSa3 (88). Allelic variation in *hlb* between hST1 and bST1 genomes may be linked to φSa3 integration, and the presence of φSa3 in a bovine isolate raises questions about MGE transmission and potential human-to-animal gene flow (81), all of which warrant further investigation.

Clade-level analysis supports long-term evolutionary separation, with higher SNP distances between hST1 and bST1 genomes in Clades 1.2 (319 SNPs) and 3 (273 SNPs). φSa3 was absent from all bovine isolates in Clade 1.2 and 3 but present in the three human genomes within Clade 3. This consistent absence in bST1 genomes, despite close phylogenetic relatedness, supports the notion of progressive host adaptation, where the loss of human-specific MGEs may benefit bovine hosts. However, we cannot date the timing of these gene loss events (7, 89–92).

SRR20727552, a bST1 genome in Clade 1.1, is a notable exception. This genome retained human-associated AMR and virulence genes and differed by only 61 SNPs from hST1 genomes, suggesting recent divergence. The most recent common ancestor of SRR20727552 dates to 1971, six years before Yebra et al. reported a CC1 human-bovine host jump in Australia around 1977 (13). This may reflect a recent spillover with limited adaptation to bovine hosts. As SRR20727552 originated from an independent New Zealand bovine *S. aureus* study (19), reverse zoonosis is plausible, but strain mix-up or contamination cannot be excluded and should be considered when interpreting this finding.

Genomic screening revealed clear differences in AMR and virulence gene composition between bST1 and hST1 isolates. Efflux pumps and regulatory genes were broadly conserved across both host populations, whereas genes such as *mecA*, *ermC*, and *fusC* were more prevalent in hST1 genomes, consistent with greater exposure to clinical antibiotics (93, 94). The *blaZ* gene was present in both host groups and found to confer resistance phenotypically to penicillin, while *tet*(38) showed no association with resistance, aligning with global findings (95). Virulence gene profiles followed similar patterns. hST1 genomes were enriched in immune evasion genes (*sak*, *scn*, and *chp*) (91, 96) and enterotoxins, while bST1 genomes possessed bovine-adapted leukocidins (*lukF-like*/*lukM*) (75, 76) alongside allelic variants of core virulence factors. Despite only 67.7% query coverage, two *blaZ*-positive isolates displayed phenotypic resistance, reinforcing the importance of retaining low-coverage hits in AMR and virulence gene screening rather than filtering them out automatically. However, *tet*(38) serves as an exception, demonstrating that genomic presence alone does not indicate resistance, emphasizing the need for functional validation when genotype-phenotype correlation remains uncertain.

Our study has several limitations. Human isolates were sourced from clinical skin and wound infections, while bovine samples were convenience samples from subclinical mastitis and bulk milk sources. Differences in disease state and anatomical origin may influence gene content and warrant further investigation (14, 93, 94, 97–99). Although we applied FDR correction, the risk of false positives from multiple testing remains. Additionally, we did not adjust for potential confounders such as geographical origin or antibiotic use, and gene co-occurrence or sample relatedness, which may affect data independence. The long branch lengths in Clade 2, along with the overrepresentation of 2014 and 2019 genomes in Clade 1, highlight the importance of more temporally diverse sampling to improve the resolution of evolutionary timelines. In particular, the low estimated mutation rate of Clade 1 may be a consequence of this skewed sampling distribution.

Overall, our findings underscore the importance of a One Health approach in monitoring *S. aureus* evolution, highlighting the interconnectedness of human, animal, and environmental health (77, 78, 100, 101). Future research should include broader sampling from livestock workers and environmental reservoirs to assess transmission dynamics and zoonotic potential. Continued surveillance of MGEs like φSabovST1 and φSa3 and functional validation of resistance and virulence genes will be essential to inform AMR stewardship and biosecurity strategies. New Zealand’s distinct epidemiological setting offers a valuable model for understanding pathogen evolution in relatively closed systems, with lessons that are increasingly relevant in a globally connected world.

### Concluding remarks

Our findings highlight the host ecology of *S. aureus* ST1 in New Zealand, shaped by resistance, virulence, and mobile genetic elements. Integrating genomic surveillance with host-specific and ecological data offers a framework for developing proactive strategies to monitor, prevent, and manage zoonotic pathogens.

## Data Availability

Short-read sequence data have been deposited in National Center for Biotechnology Information BioProject PRJNA1130542, and the accession numbers are detailed in Tables S1 and S2. The complete genome assembly for strain 23EV612 and φSabovST1 prophage has been deposited in GenBank under accession numbers CP160024 and PQ281812.1, respectively. All data processing and analysis scripts used in this study are available on GitHub at https://github.com/emv6/Comparative_Genomics_ST1_Staphylococcus_aureus/.

## SUPPLEMENTAL MATERIAL

Supplemental Material msphere.00376-25-s0001.pdf Experimental details and Fig. S1-S15.

Supplemental Tables msphere.00376-25-s0002.xlsx Tables S1-S20.

ASM does not own the copyrights to Supplemental Material that may be linked to, or accessed through, an article. The authors have granted ASM a non-exclusive, world-wide license to publish the Supplemental Material files. Please contact the corresponding author directly for reuse.
