# Supplementary material for: Comparative genomics of endemic Staphylococcus aureus ST1 in New Zealand
Source: mSphere. 2025 Sep 23;10(10):e00376-25. doi: 10.1128/msphere.00376-25 (PMC12570506; doi:10.1128/msphere.00376-25)
Supplement: Supplemental Material — Experimental details and Fig. S1-S15. [file msphere.00376-25-s0001.pdf]

Supplementary Materials for Comparative Genomics of Endemic ST1 *Staphylococcus aureus* in New Zealand

Emma M Voss[1,2#], Gregory M. Cook[2,3], Christine Couldrey[1*], Scott A. Ferguson[2], Chad Harland[1], Ali Karkaba[4,5], Scott McDougall[4], Sergio E. Morales[2,6], Jack Rolfe[7], James E. Ussher[2], Rhys. T. White[8], Liam Williams[7] and John Williamson[1]

[1]Livestock Improvement Corporation (LIC) –Research and Development –Newstead, New Zealand

[2]Department of Microbiology and Immunology, University of Otago, Dunedin, New Zealand.

[3]Queensland University of Technology, School of Biomedical Sciences, Brisbane, Queensland, Australia

[4]Cognosco, Anexa Veterinary Services, Morrinsville, New Zealand

[5]American International University, Kuwait

[6]MPG Ranch, Missoula, MT, USA

[7]Livestock Improvement Corporation (LIC) – Diagnostics – Riverlea, New Zealand

[8]New Zealand Institute for Public Health and Forensic Science, Health Security, Porirua, New Zealand

#Address correspondence to Emma M. Voss, Emma.Voss@lic.co.nz

*Present Address: Christine Couldrey, Independent Science Consultant, Hamilton, New Zealand.

This file includes the following:

Supplementary Methods

Figure S1: Graphical Representation of Sample Collection

Figure S2: New Zealand map depicting regions

Figure S3: Presence/absence heatmap for detected adherence virulence genes

Figure S4: Presence/absence heatmap for detected enterotoxin virulence genes

Figure S5: Presence/absence heatmap for detected exoenzyme virulence genes

Figure S6: Presence/absence heatmap for detected exotoxin virulence genes

Figure S7: Presence/absence heatmap for detected haemolysin virulence genes

Figure S8: Presence/absence heatmap for detected immune modulation virulence genes

Figure S9: Presence/absence heatmap for detected intracellular adhesion virulence genes

Figure S10: Presence/absence heatmap for detected Type VII secretion system virulence genes

Figure S11: Presence/absence heatmap for detected genes classed in a determined "Other" category

Figure S12: Maximum likelihood phylogeny of 520 ST1 *S. aureus* strains and temporal signal

Figure S13: Maximum likelihood phylogeny of Clade 1 ST1 and temporal signal

Figure S14: Maximum likelihood phylogeny of Clade 2 ST1 and temporal signal

Figure S15: 23EV612 genome visualisation with bacteriophage φSabovST1 location

Supplementary Methods

Culture of Bovine *S. aureus* isolates

Between July 2019 and November 2023, bovine *S. aureus* isolates were cultured from a bulk milk tank (n=618), foremilk aseptic (n=305) and composite milk samples (n=5) from across New Zealand (Figure S1). Many samples were collected for animal health testing and trials conducted by Livestock Improvement Corporation (LIC), a herd improvement and agri-technology company. Ethics approval was not required for bulk tank milk sampling; however, ethics approval for the individual quarter foremilk and composite milk samples was obtained from the Ruakura Animal Ethics Committee, Hamilton, North Island (Approval Numbers: 14240, 15410 and 15575).

A bulk tank sample is a composite sample of milk from all lactating cows in a herd that are milked and collected in a central storage tank, commonly known as a "bulk tank" (Figure S1 A-B). The bulk tank samples were collected in 35 mL sample containers as part of LIC animal health testing and a LIC nationwide bulk tank trial for the research program MilkOmics®. Farmers across New Zealand voluntarily enrolled in the MilkOmics® trial under the condition that they herd-tested with LIC at least once during the milking season, unless prior arrangements were made. The trial aimed to collect representative milk samples from a broad geographic distribution across the country. For animal health testing, tanker drivers collected the 35 mL milk samples, while for MilkOmics®, farmers collected the 35 mL milk samples containing 0.1 mL of bronopol preservative. Containers were delivered to the LIC Animal Health Laboratory, Hamilton, North Island, New Zealand.

Foremilk aseptic quarter and composite milk samples were collected in three LIC research trials. Two trials involved selecting cows with SCC counts of over >200,000 cells/mL in a recent production milk sample. The focus of the third trial was the longitudinal sampling of ten cows for eight months (September 2021 - April 2022). Aseptic quarter milk samples were

collected before milking (hence the name foremilk). The cows selected for sampling were drafted into the milking shed, and each cow's teat was cleaned with 70% ethanol-soaked cotton pads to remove any faecal or dirt matter before hand milking using strict hygienic (aseptic) procedures (sterile equipment, hand hygiene and disinfection) [1]. The teats closest to the sampler were milked first to avoid the sampler's arms contaminating clean teats. Each quarter (teat) of each cow was milked separately into a collection container (Figure S1 C) [1]. A composite sample is the foremilk aseptic sample from each quarter pooled into a single collection container; five cows had their quarters pooled to form composite samples (Figure S1D). Farms across New Zealand were selected for inclusion in the three trials based on a prior history of *S. aureus* infection, as identified by veterinarians or participation in previous research trials. Animal health technicians collected aseptic quarter milk samples within the North Island, while veterinarians collected South Island milk samples. Containers were delivered to the LIC Animal Health Laboratory, Hamilton, North Island, New Zealand.

All consumables, unless stated, were sourced from Fort Richard Laboratories, Auckland, North Island, New Zealand. Bacteriology was completed to determine the presence of *S. aureus*. 100 µL of bulk tank milk was spread-plated onto esculin sheep blood agar, and plates were incubated for 48 hr at 37°C. For aseptic quarter/composite milk samples, the milk volume was reduced to 10 µL. Suspected *S. aureus* colonies were identified as having a positive rabbit plasma coagulase test, positive catalase and incomplete or complete haemolysis zones [2]. All positive *S. aureus* isolates were re-streaked twice on esculin sheep blood agar to ensure culture purity before placing two to three colonies into Laboratoire de Santé Publique du Québec (LSPQ) preservation medium and stored at -20 °C before DNA extraction. We cultured 216 isolates from the foremilk of 136 cows. In 46 cows, isolates were obtained from more than one quarter. Three of the ten cows selected for monthly sampling had *S. aureus* isolates detected in one or more quarters across consecutive months. *S. aureus* isolates were cultured from all five

composite samples. From 231 farms, 618 *S. aureus* isolates were cultured from bulk tank milk. It is important to note that 141 farms had multiple *S. aureus* isolates recovered in one bulk tank milk sample and/or *S. aureus* isolates cultured from the same farm over the milking season.

In addition to our *S. aureus* isolates described above, we received 89 *S. aureus* isolates cultured from aseptic quarters from two sources: 29 isolates from a 2005 DairyNZ/LIC Taranaki farm trial and 60 isolates from routine diagnostic work conducted at Anexa Veterinary Services, Morrinsville (Cognosco). We reconfirmed the identification of *S. aureus* as described above. 310 *S. aureus* isolates were cultured from foremilk aseptic quarter/composite samples and 618 from bulk tank milk, resulting in an overall total of 928 isolates for sequencing.

Broth DNA Isolation & Extraction

S. aureus isolates (n=928) were re-streaked onto esculin sheep blood agar from LSPQ stock, and agar plates were incubated at 37°C for 24 hr. A 10 µL loop of a single *S. aureus* colony was added to 2 mL of Tryptic Soy Broth (TSB) and incubated at 37°C for 17 hr. In addition, *S. aureus* ATCC 25923 was cultured and incubated with TSB to serve as a positive control, and one Falcon tube contained TSB only to serve as a blank. The bacterial-liquid suspension was centrifuged for 5 min at 3200 RCF, the supernatant was discarded, and the resulting pellet was resuspended in 500 µL of sterile Phosphate-Buffered Saline. Each *S. aureus* isolate was transferred to a well in a 96-well plate for DNA extraction.

Genomic DNA was extracted using a Kingfisher machine (ThermoFisher, New Zealand) and a BioSprint® 96 DNA kit (Qiagen) with a bead-beating step. The DNA concentrations were measured following the manufacturer's protocol for the 1× dsDNA Broad Range Assay Kit (ThermoFisher, New Zealand). All isolates yielded DNA concentrations >30 ng/µL, and negative controls (TSB) registered a negative result. The DNA was stored at -20°C.

Illumina Sequencing

S. aureus (n=928) isolates underwent Illumina sequencing, generating 150 bp paired-end sequencing reads. The targeted depth of coverage was 75-fold for each *S. aureus* isolate. Due to the timing of sample collection, sequencing libraries were prepared in batches.

In August 2020, *S. aureus* isolates (n=96) were sent to Annoroad Gene Technology Corporation, Beijing, China. Following the manufacturer's recommendations, sequencing libraries were generated using NEBNext® Ultra™ DNA Library Prep Kit for Illumina (NEB, USA), and index codes were added to attribute sequences to each sample. Each indexed library was pooled and sequenced on an Illumina Novaseq 6000 (Illumina, USA).

In November 2021, 65 *S. aureus* isolates had libraries prepared using an Illumina DNA Preparation Kit and Illumina UD Indexes (Plate A/Set1, Plate B/Set2, Plate C/Set3, Plate D/Set4) (Integrated DNA Technologies (IDT), USA). Sequencing was completed in-house at LIC (Riverlea, Hamilton, New Zealand) on a Novaseq 6000 (Illumina, USA) with an S1 flow cell. In June 2022 and March 2023, sequencing libraries were prepared in-house at LIC (Riverlea, Hamilton, New Zealand) (n=709) using Illumina DNA Preparation Kit and Illumina UD Indexes (Plate A/Set1, Plate B/Set2, Plate C/Set3, Plate D/Set4) (Integrated DNA Technologies (IDT), USA). Sequencing was completed on an Illumina Novaseq 6000 on an S1 flow cell, utilising XP loading.

The remaining 58 *S. aureus* genomes were sent to Annoroad Gene Technology Corporation, Beijing, China, in April 2024. Following the manufacturer's instructions, sequencing libraries were prepared using NEBNext® Ultra™ DNA Library Prep Kit for Illumina (NEB, USA), and index codes were added to attribute sequences to each sample. Clustering of the index-coded samples was performed on a cBot Cluster Generation System according to the

manufacturer's instructions before sequencing was carried out on an Illumina Novaseq X Plus (Illumina, USA).

Hybrid Genome Assembly for 23EV612

Isolate DNA for nanopore sequencing was obtained from the same extraction plate used for Illumina sequencing. In July 2021, sequencing libraries for 23EV612 and an additional *S. aureus* isolate were prepared using the Native Barcoding Amplicon protocol (EXP-NBD104 & SQK-LSK109) following the manufacturer's instructions (NBA_9093_v109_revD_12Nov2019). The two isolates were pooled for sequencing on a R.9.4.1 MinION flow cell with 1200 pores on an MK1C device using fast base-calling and demultiplexing using Guppy v6.0.1 [3]. The sequencing was run for 28 hr, generating 1.6 million reads. The genome assembly process is detailed in the Hybrid_Genome_Assembly.sh file on https://github.com/emv6/Comparative_Genomics_ST1_Staphylococcus_aureus. Guppy v6.4.6 [4] was used to base call all nanopore reads using the super accurate model. Reads greater than 1000 bp and the top 95% of reads were retained using Filtlong v0.2 (https://github.com/rrwick/Filtlong, accessed on 1 March 2022). Barcodes and adaptors were removed using Porechop v0.2.4 (https://github.com/rrwick/Porechop, accessed on 1 March 2022). NanoStat v1.5 and Chopper v0.20 [5] were used to determine the mean read quality and filter out reads with a quality below 17.8. Flye v2.9.1 [6] was used as the *de novo* long-read assembly, and the final assembly was polished using medaka v1.6.0 (https://github.com/nanoporetech/medaka, accessed on 18 March 2022). Circlator v1.5.5 [7] was used with default settings to circulate the assembly. Illumina reads were mapped to the nanopore assembly using BWA v0.7.17 [8] and SAMtools v1.16.1 [9]. Three iterations of Pilon v1.2.4 [10] were performed to polish the hybrid assembly. The assembly was evaluated using CheckM v1.2.1 [11] and Quality Assessment Tool (QUAST) v5.2.0 [12]. The resistome and virulence genes were annotated using ABRicate v1.0.1

(https://github.com/tseemann/abricate, accessed 18 March 2022). The resistome database was specified as Comprehensive Antibiotic Resistance Database (CARD) v3.2.9 [13] and virulome identification was completed using the Virulence Factor Full Database (VFDB) - accessed 15 March 2024 [14].

Temporal Analysis using Bayesian ancestral state reconstruction using BEAST v2.7.7

To explore the temporal signal for molecular clock analyses, we initially assessed root-to-tip divergence against sampling dates using TempEst v1.5.3 [15]. The *S. aureus* ST1 phylogeny revealed two distinct clusters, hereafter designated Clade 1 and Clade 2. To further explore potential lineage-specific temporal structure, the clades were analysed separately. Core-genome alignments were generated for each clade using snippy v4.6.0 (https://github.com/tseemann/snippy, accessed 2nd February 2025), with Clade 1 aligned to the best-quality short-read assembly identified as a human clinical isolate H8195 (SRA: SRR29758955), and Clade 2 aligned to 23EV612 (the bovine hybrid genome; GenBank: CP160024). Recombination regions were identified and removed using Gubbins v3.2.2 [16] and snp-sites v0.8.2 (https://github.com/sanger-pathogens/snp-sites, accessed 2nd February 2025) was used to extract variant positions from the resulting alignments. Root-to-tip divergence was then re-examined in TempEst for both clades (Figure S13 & S14). Genomes that exhibited substantial deviations from the regression line, indicating a poor fit to the molecular clock model [17] were excluded from subsequent time-calibrated phylogenetic analysis (Table S9).

For both clades, a best-fit model was determined using Bayesian ancestral state reconstruction using BEAST2 v2.7.7 [18, 19]. In the Bayesian method, we initially assessed whether the strict or optimised relaxed uncorrelated clock model is more suitable for our dataset. The initial

models were created using tip dates, a GTR substitution model, and a coalescent prior with a constant population. Both models were tested with the Nested Sampling Bayesian computation algorithm v1.1.0 within the BEAST2 package with a particle count of 32, sub-chain length of 5000 and Epsilon of 1.0×10^{-12}. This analysis supports the use of an uncorrelated relaxed clock model for Clade 1 [20, 21], which yielded higher marginal likelihoods than the strict clock model: relaxed clock = -18,526.30 (standard deviation (SD): ±4.02) vs. strict clock = -18,537.36 (SD: ±4.00). For Clade 2, a strict clock was supported [18], with a higher marginal likelihood compared to the uncorrelated relaxed clock: strict clock = -184,990.93 (SD: ±4.45) vs. relaxed clock = -186,817.47 (SD: ±4.60).

The Bayesian skyline, coalescent constant, and exponential growth population size change models were evaluated for the optimised relaxed uncorrelated clock model. The Gamma Site Model Category Count was set to four, and the GTR substitution model rates determined from jModelTest v2.1.10 [22] were applied: for Clade 1 - AC=1.06, AG=3.92, AT=1.31, CG=0.31, CT=3.76, and GT=1.00; and for Clade 2 - AC=0.97, AG=3.48, AT=1.27, CG=0.45, CT=3.51, and GT=1.00. The initial clock rate was set to the rate estimated from the root-to-tip regression analysis in TempEst (i.e., 7.82×10^{-6} substitutions per site per year for Clade 1 and 6.66×10^{-6} for Clade 2; (Figure S13 & S14) with a uniform distribution and an upper bound of 0.1. All other priors were left as default. We performed three independent Markov-chain Monte Carlo runs using 20 million generations for each analysis. Trees were sampled every 1,000 generations, resulting in a triplicate sample of 20,000 trees for each model test (Table S10 & S11). To evaluate the metrics, all BEAST2 executions were loaded into Tracer v1.7.2 (http://github.com/beast-dev/tracer/, accessed on 14 November 2024). LogCombiner v2.7.7 (part of the BEAST2 package) subsequently combined the replicated analyses for each model with a 10% burn-in to evaluate convergence.

After identifying the most suitable tree model, three separate Markov chain Monte Carlo runs were performed for 100 million generations for each analysis. Trees were collected every 1,000 generations, yielding three sets of 100,000 trees. TreeAnnotator v2.7.7 (part of the BEAST2 package) produced maximum clade credibility trees from each run (derived from 270,000 trees), annotating median values with a posterior probability limit set at 0.5. The phylogenetic trees produced were visualised in FigTree v1.4.4 (http://tree.bio.ed.ac.uk/software/figtree/, accessed 05 November 2024) and TreeViewer [23].

Supplementary Figures

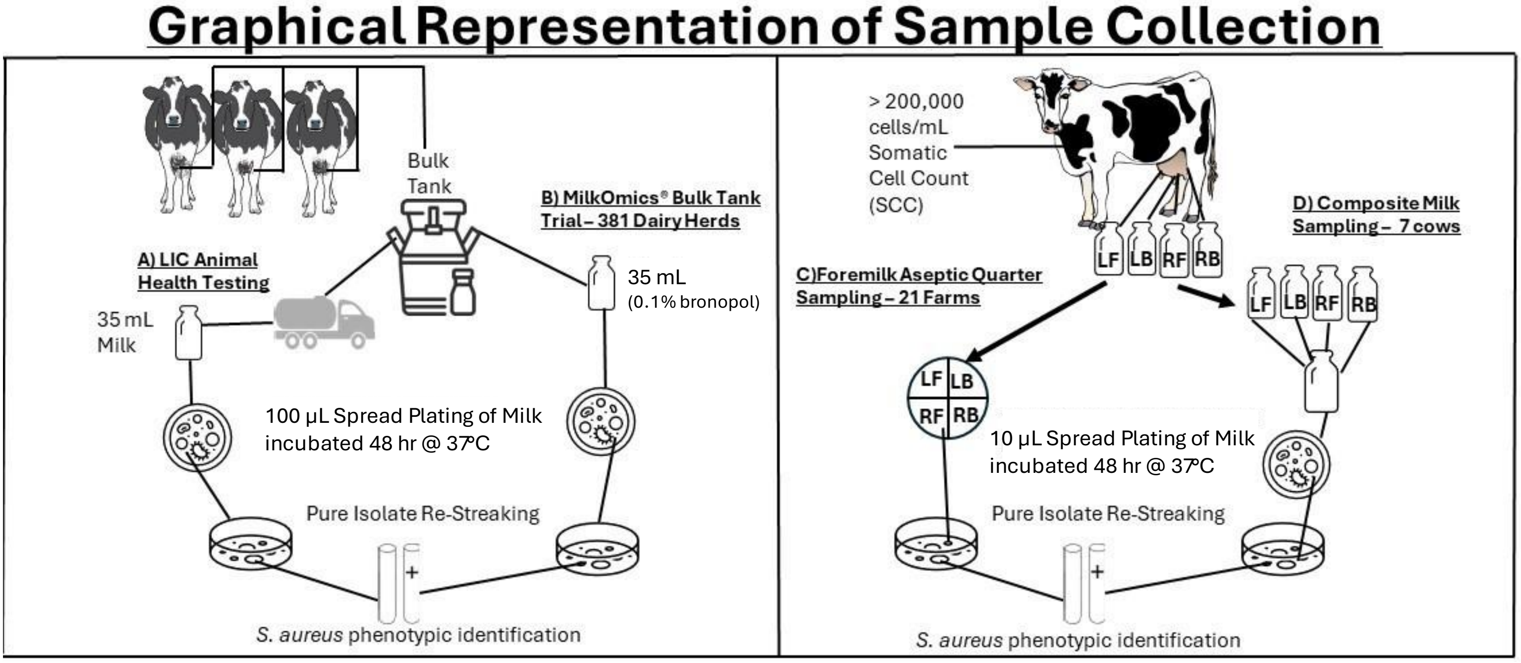

Figure S1: Graphical Representation of Sample Collection. A and B (left panel) depict the process for collecting milk samples referred to as "bulk tank milk samples". Milk is collected from each cow's udder via a milking cluster, which is transferred to a bulk tank collection tank. Milk is stored in the bulk tank until collection and transported to a dairy processing plant. A bulk tank milk sample is a composite (combined) sample of all lactating milk cows in the herd/farm that are being milked for supply on the day of collection. A 35 mL sample from the bulk tank is collected, taken to the laboratory, and plated onto esculin sheep blood agar to culture bacteria. In the MilkOmics® bulk tank trial, the 35 mL milk sample contains 0.1 % bronopol, a preservative for long-term milk storage. **C and D (right panel)** depict the process for foremilk aseptic quarter-level sampling and cow-composite milk sampling. Aseptic quarter samples are collected before milking, hence the name foremilk. Cows with an elevated somatic cell count (SCC) > 200,000 cells/mL, indicate infection and cows with this SCC are selected for sampling. Each cow's teat is cleaned with an ethanol wipe, and a standard aseptic sampling technique is followed to collect a milk sample from each quarter (teat) into a collection container. A composite sample is a foremilk aseptic sample from each quarter pooled into one collection container. Phenotypic methods – positive rabbit plasma coagulase, positive catalase and incomplete/complete haemolysis are used to identify *S. aureus* before storing each isolate into a charcoal glycerol-based media (LSPQ) commonly used to store milk isolates. The icons and images are sourced from https://ian.umces.edu/media-library/symbols/ and stock icons in Microsoft Word accessed 20th March 2024.

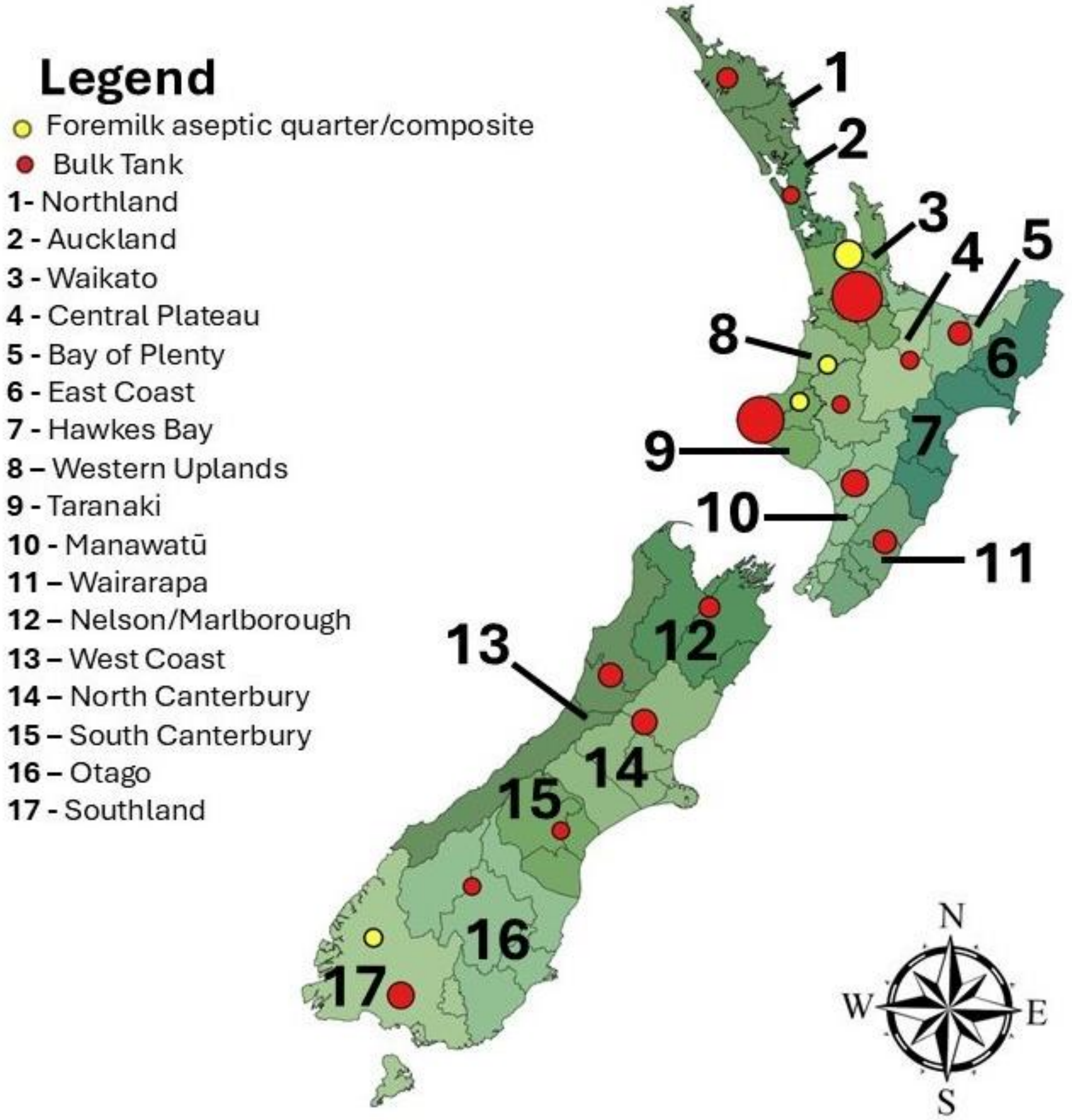

Figure S2: New Zealand map depicting regions defined by the New Zealand Dairy Statistics and coloured dots depicting the count of bovine ST1 *S. aureus* isolates collected from each region from this study. Each dot represents the total quantity of *S. aureus* isolates collected from bulk tank milk samples (red) and foremilk aseptic quarter samples (yellow) in the fifteen sampled regions. The dot size is correlated to the quantity of *S. aureus* isolates were collected, with a higher density of isolates collected from the Waikato (3) and Taranaki (5) regions. No bovine *S. aureus* isolates from trial work have been collected from region 6 (East Coast) and region 7 (Hawkes Bay). The map has been generated in ArcGIS Pro 3.1.0 utilising NZ Dairy Statistics [24, 25].

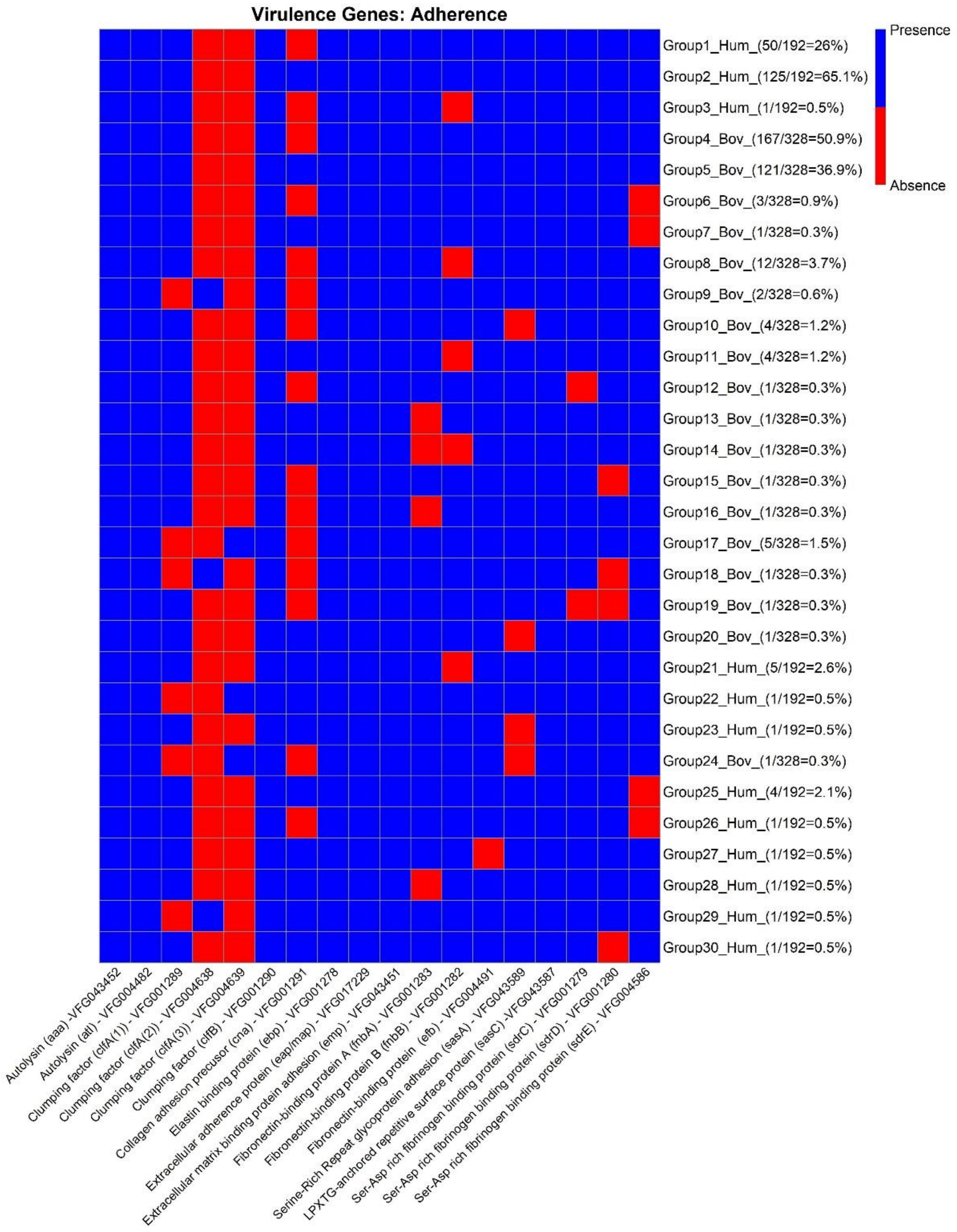

Figure S3: Presence/absence heatmap for detected adherence virulence genes generated by *pheatmap* in R [26]. Genes are labelled at the bottom of each heatmap. The presence of the gene is depicted in blue, with the absence of the gene in red. Groups have been generated based on each gene's presence/absence profile and the host of each *S. aureus* isolate (https://github.com/emv6/Comparative_Genomics_ST1_Staphylococcus_aureus/blob/main/AdherenceGroup.csv).

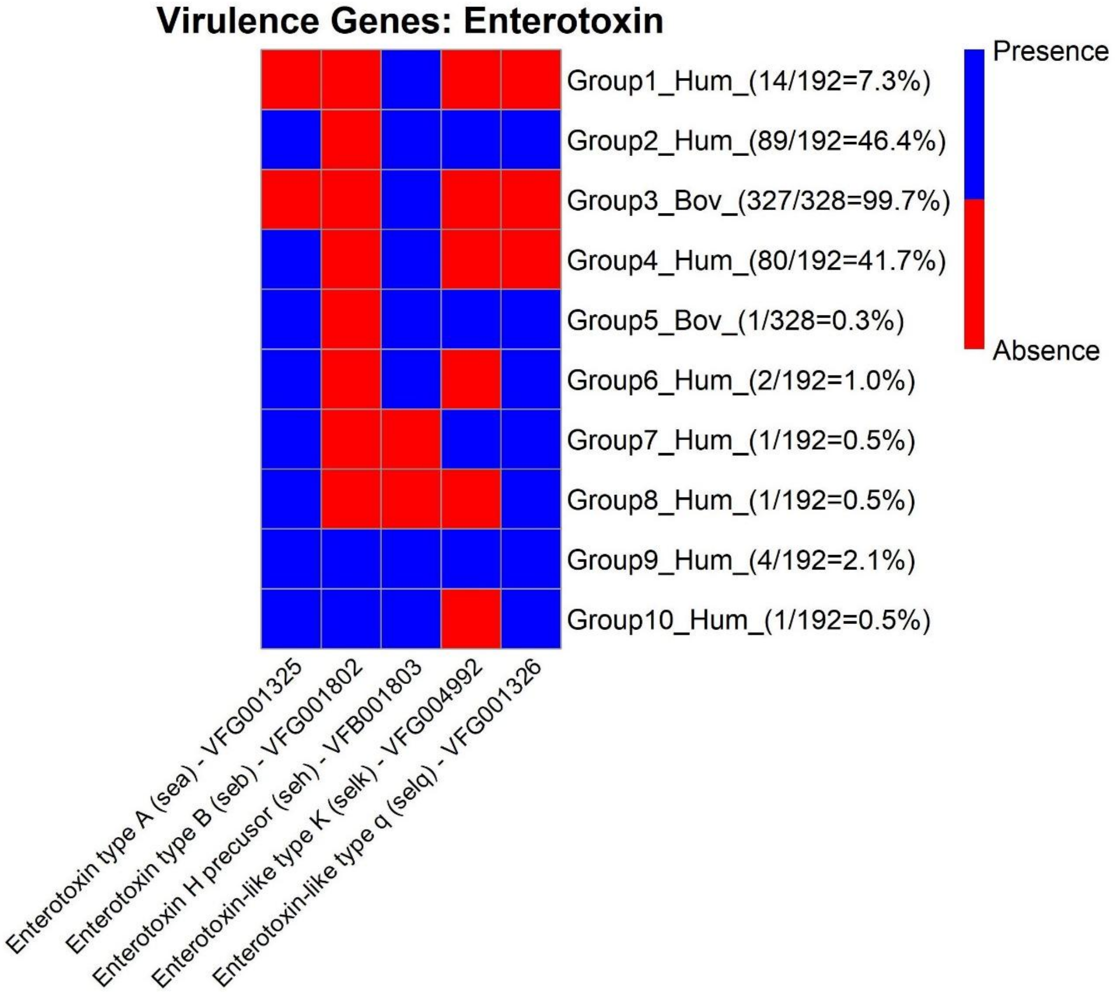

Figure S4: Presence/absence heatmap for detected enterotoxin virulence genes generated by *pheatmap* in R [26]. Genes are labelled at the bottom of each heatmap. The presence of a gene is depicted in blue, and its absence is in red. Groups have been generated based on each gene's presence/absence profile and the host of each *S. aureus* isolate (https://github.com/emv6/Comparative_Genomics_ST1_Staphylococcus_aureus/blob/main/enterotoxinGroup.csv).

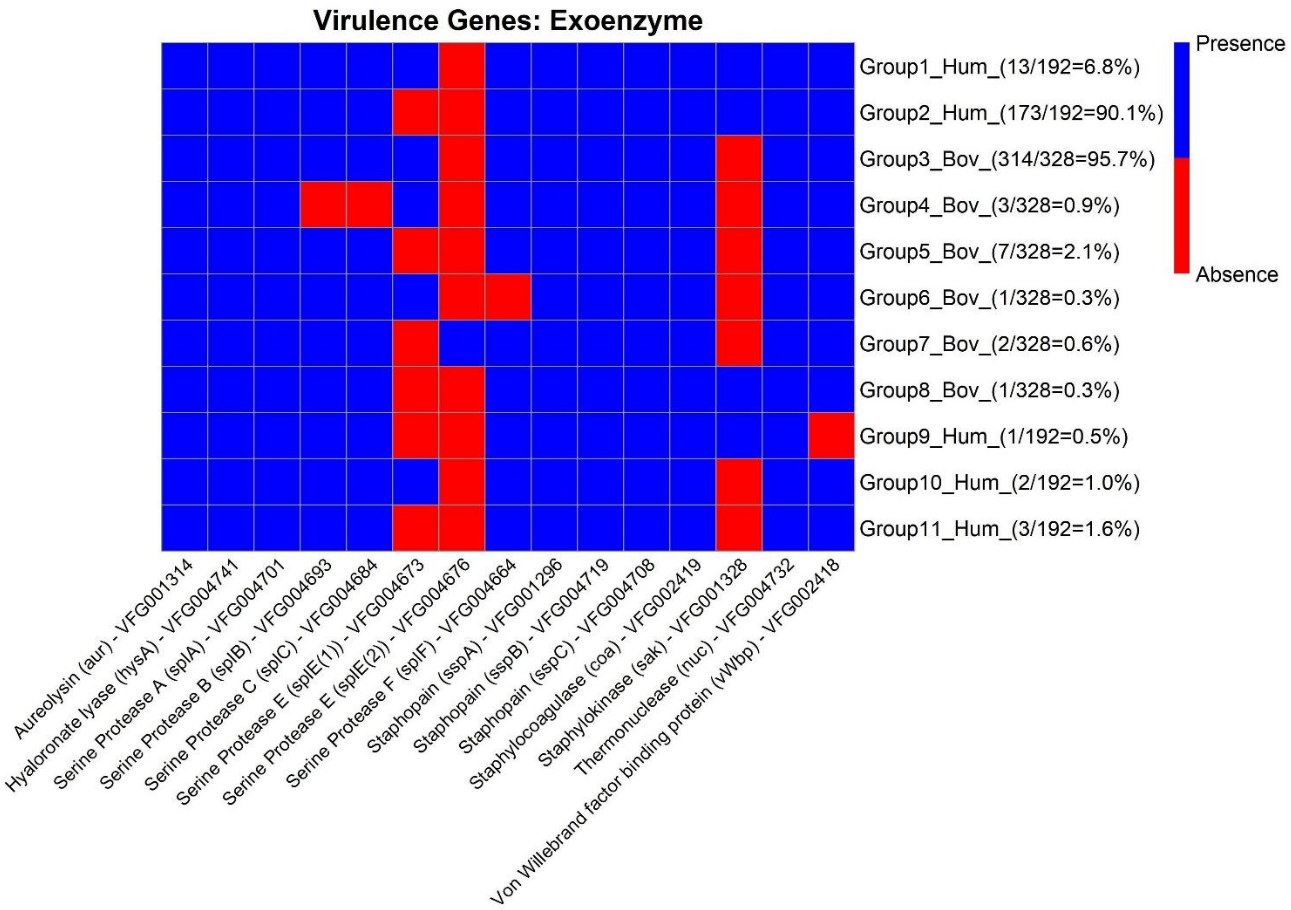

Figure S5: Presence/absence heatmap for detected exoenzyme virulence genes generated by *pheatmap* in R [26]. Genes are labelled at the bottom of each heatmap. The presence of the gene is depicted in blue, with the absence of the gene in red. Groups have been generated based on each gene's presence/absence profile and the host of each *S. aureus* isolate (https://github.com/emv6/Comparative_Genomics_ST1_Staphylococcus_aureus/blob/main/ExoenzymeGroup.csv).

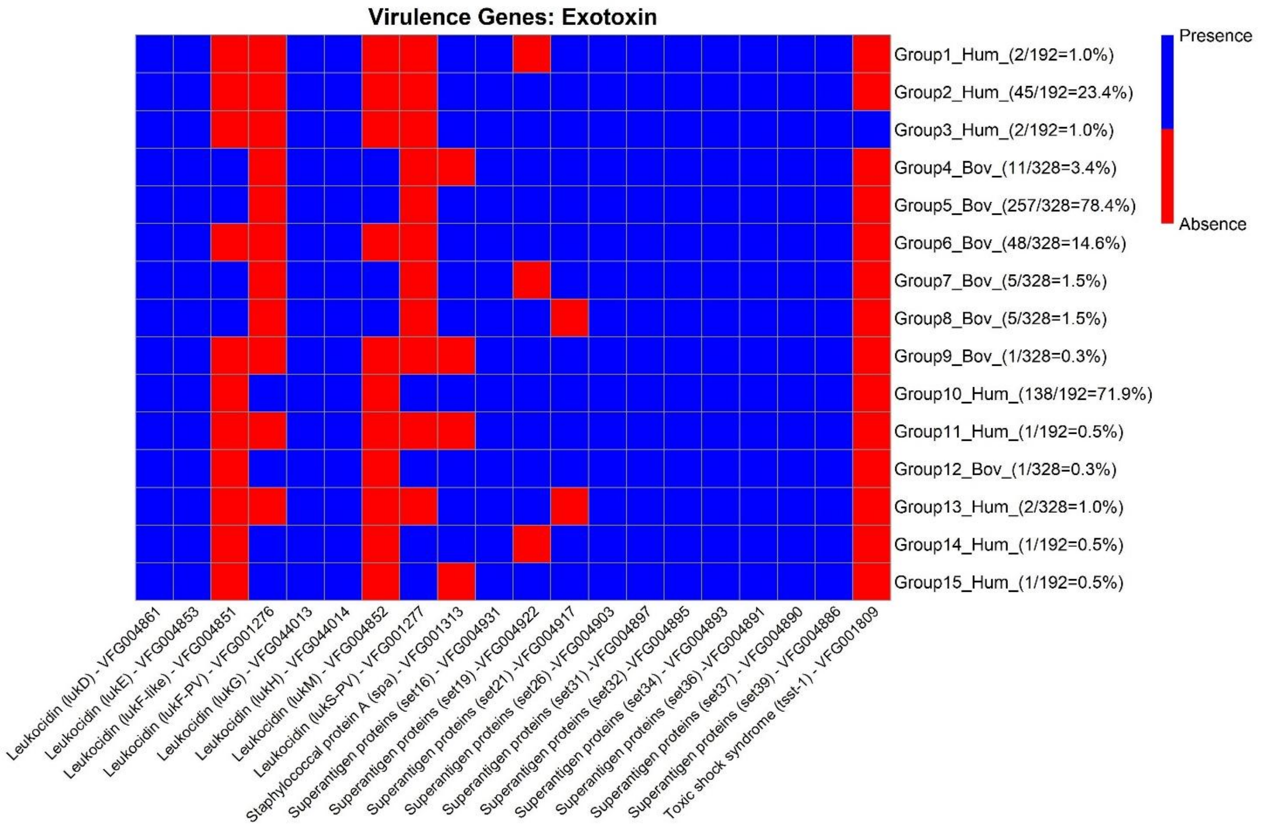

Figure S6: Presence/absence heatmap for detected exotoxin virulence genes generated by *pheatmap* in R [26]. Genes are labelled at the bottom of each heatmap. The presence of the gene is depicted in blue, with the absence of the gene in red. Groups have been generated based on each gene's presence/absence profile and the host of each *S. aureus* isolate (https://github.com/emv6/Comparative_Genomics_ST1_Staphylococcus_aureus/blob/main/ExotoxinGroup.csv).

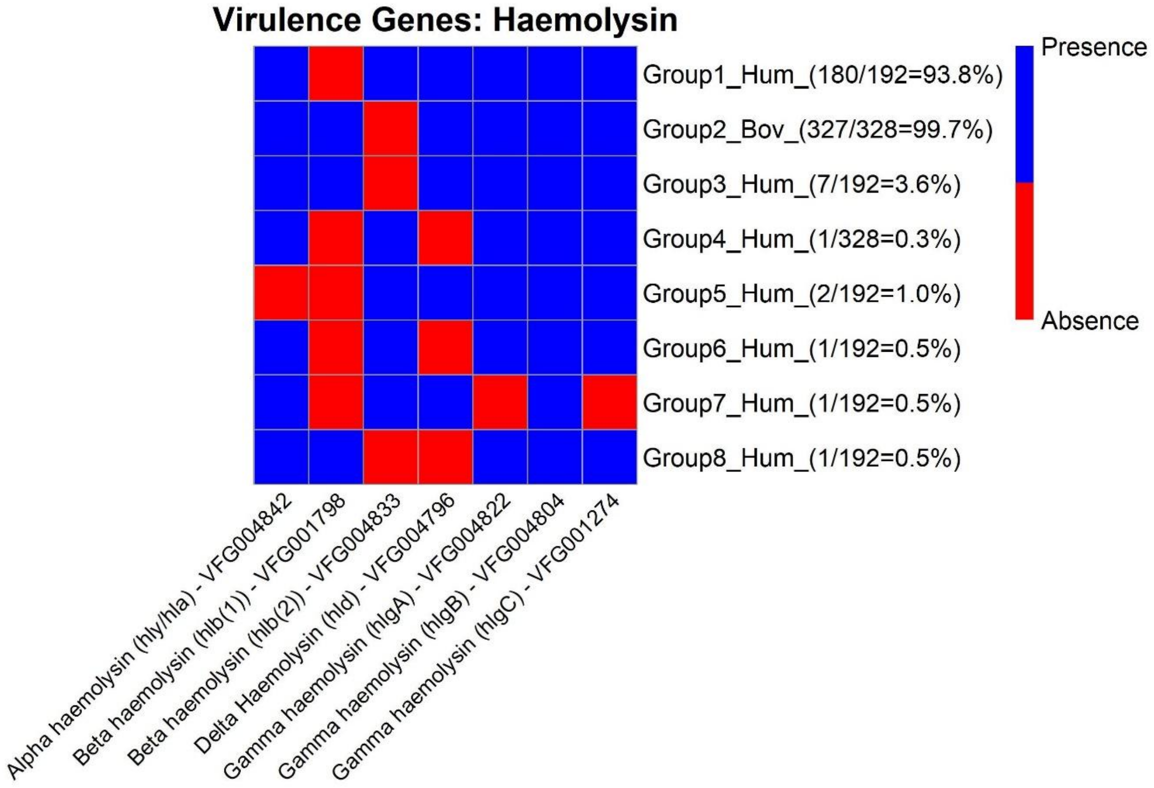

Figure S7: Presence/absence heatmap for detected haemolysin virulence genes generated by *pheatmap* in R [26]. Genes are labelled at the bottom of each heatmap. The presence of the gene is depicted in blue, with the absence of the gene in red. Groups have been generated based on each gene's presence/absence profile and the host of each *S. aureus* isolate (https://github.com/emv6/Comparative_Genomics_ST1_Staphylococcus_aureus/blob/main/haemolysinGroup.csv).

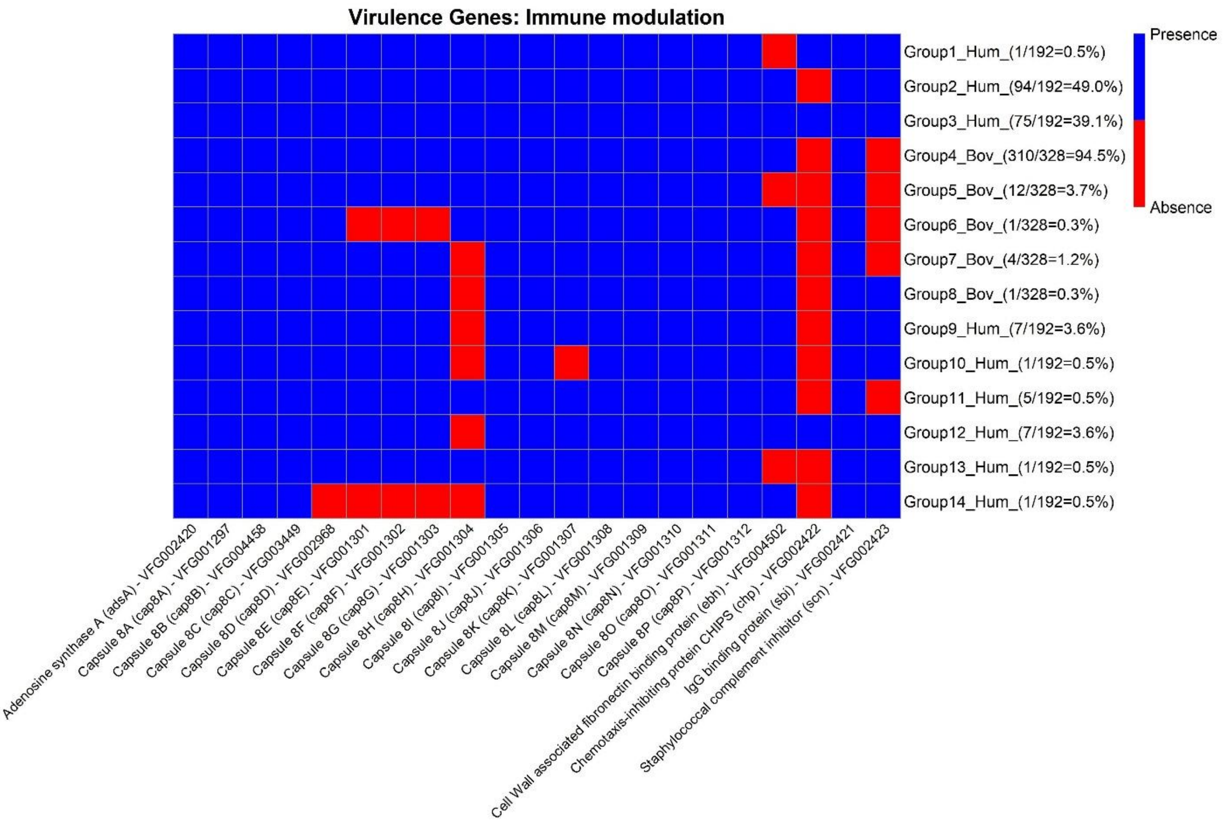

Figure S8: Presence/absence heatmap for detected immune modulation virulence genes generated by *pheatmap* in R [26]. Genes are labelled at the bottom of each heatmap. The presence of a gene is depicted in blue, and its absence is in red. Groups have been generated based on each gene's presence/absence profile and the host of each *S. aureus* isolate (https://github.com/emv6/Comparative_Genomics_ST1_Staphylococcus_aureus/blob/main/Immune_modulation_Group.csv).

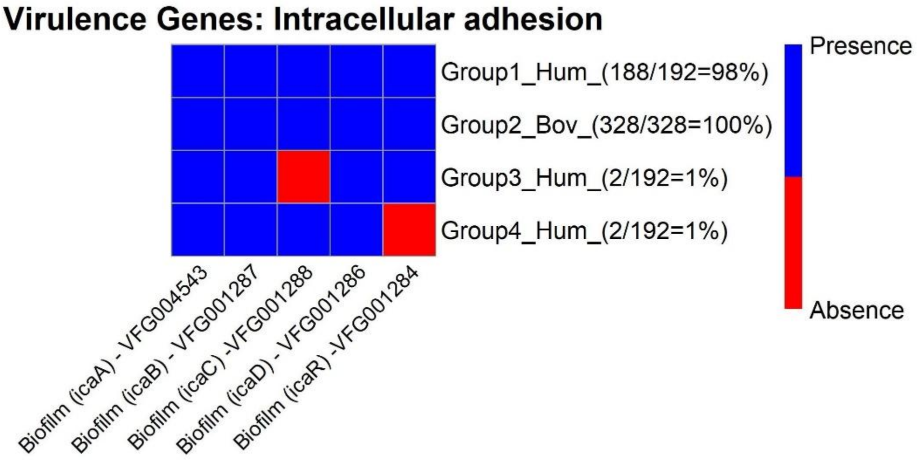

Figure S9: Presence/absence heatmap for detected intracellular adhesion virulence genes generated by *pheatmap* in R [26]. Genes are labelled at the bottom of each heatmap. The presence of the gene is depicted in blue, with the absence of the gene in red. Groups have been generated based on each gene's presence/absence profile and the host of each *S. aureus* isolate. (https://github.com/emv6/Comparative_Genomics_ST1_Staphylococcus_aureus/blob/main/Intracellular_adhesion_Group.csv)

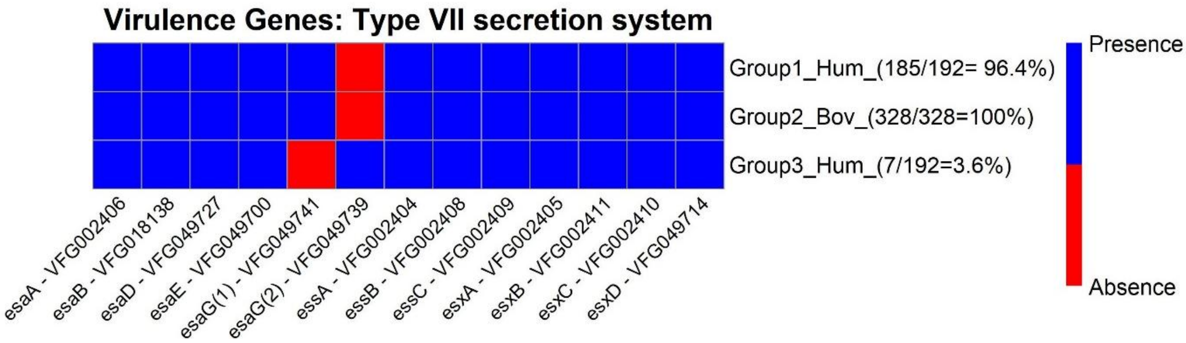

Figure S10: Presence/absence heatmap for detected Type VII secretion system virulence genes generated by *pheatmap* in R [26]. Genes are labelled at the bottom of each heatmap. The presence of the gene is depicted in blue, with the absence of the gene in red. Groups have been generated based on each gene's presence/absence profile and the host of each *S. aureus* isolate.

(https://github.com/emv6/Comparative_Genomics_ST1_Staphylococcus_aureus/blob/main/VIISecretionSystemGroup.csv).

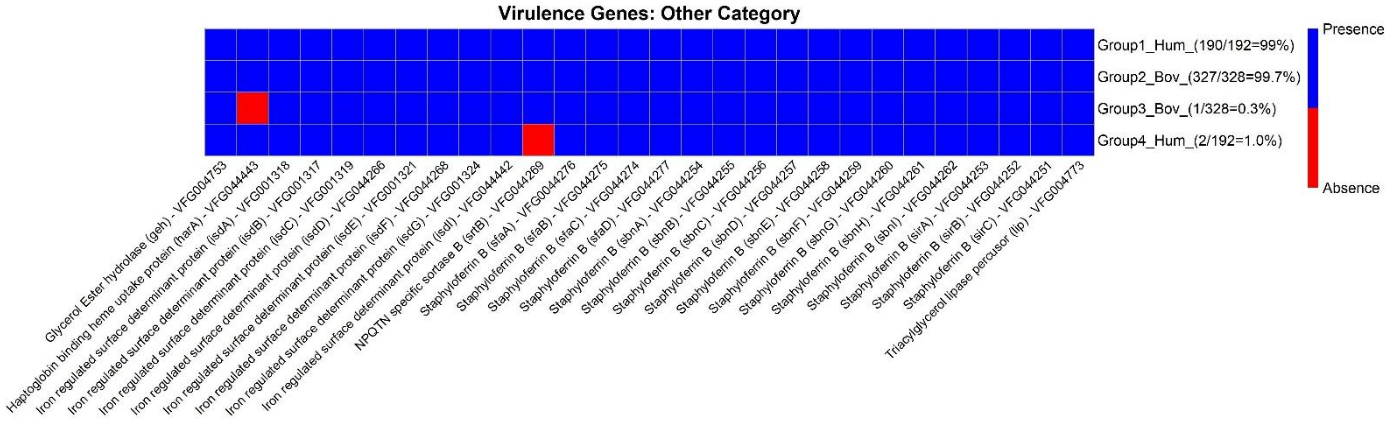

Figure S11: Presence/absence heatmap for detected genes classed in a determined 'Other' Category generated by *pheatmap* in R [26]. Genes are labelled at the bottom of each heatmap. The presence of the gene is depicted in blue, with the absence of the gene in red. Groups have been generated based on each gene's presence/absence profile and the host of each *S. aureus* isolate. (https://github.com/emv6/Comparative_Genomics_ST1_Staphylococcus_aureus/blob/main/OthersGroup.csv).

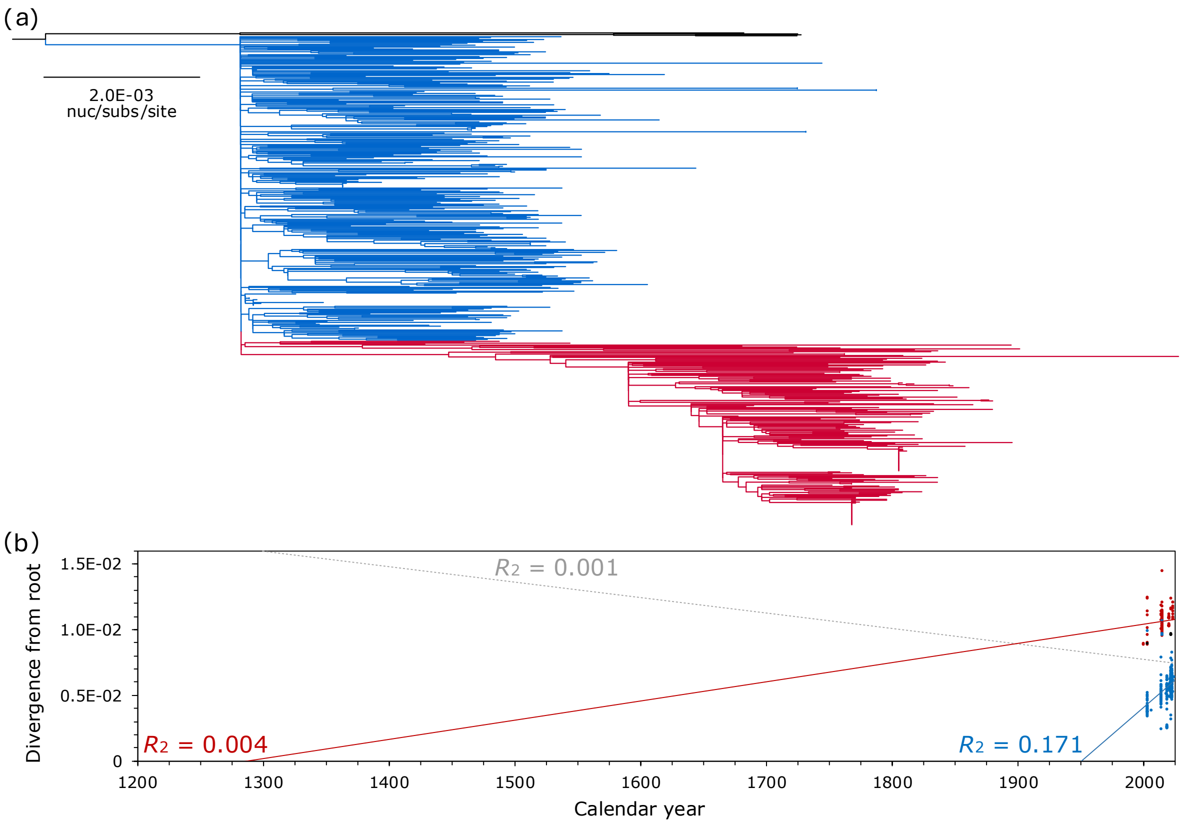

Figure S12: Maximum likelihood 520 *S. aureus* ST1 strain phylogeny (a) and temporal signal determined from TempEst 1.5.3 [15] (b). Phylogeny was determined from 19,953 variant sites rooted at the outgroup SRRR20727551 in Clade 3 (black). Clade 1 is coloured in red, and Clade 2 is coloured in blue **(a)**. The temporal signal was investigated for the 520 ST1 strain phylogeny. The linear regression for the 520 ST1 phylogeny can be seen with a low R^2 (0.001) and is dated in the future. The relationship is not clean as seen by the separation of the two clade groups (Clade 1 – red, Clade 2 – blue). Combining these two circulating clades into a single tree, the phylogeny lacks a molecular clock **(b)**.

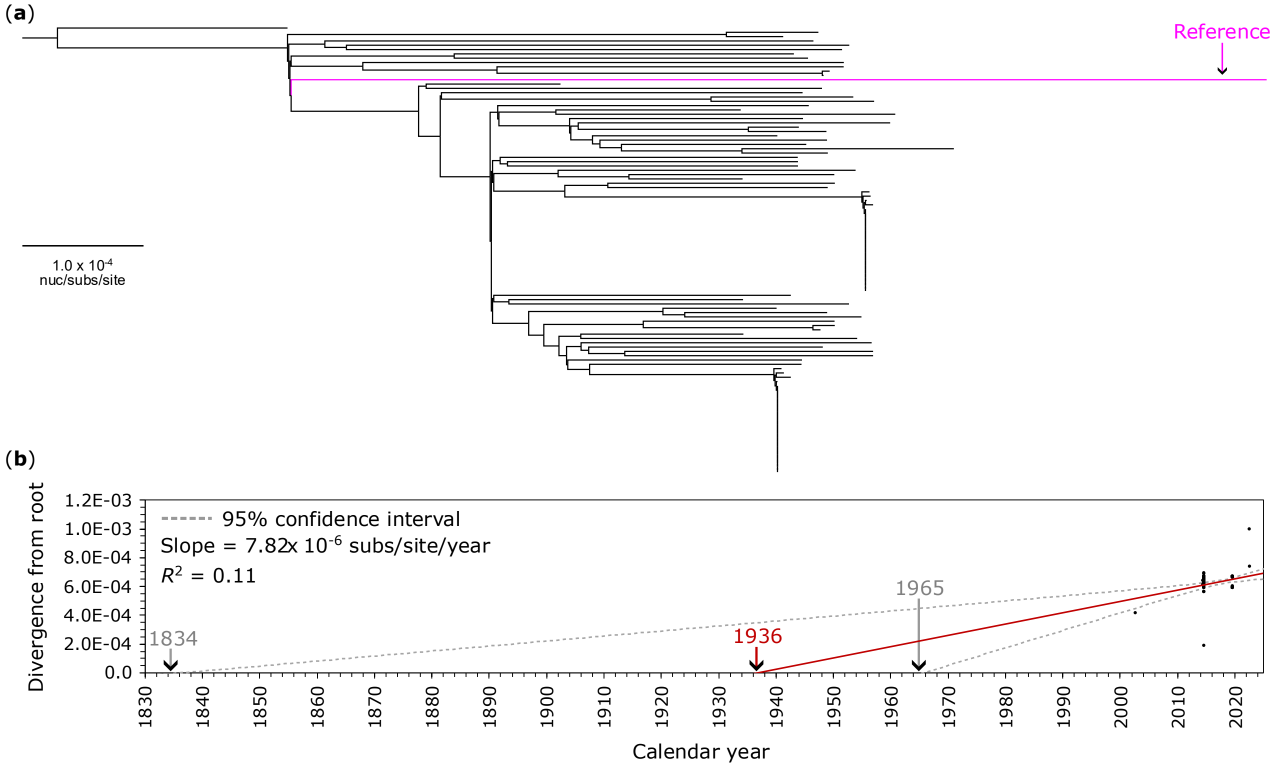

Figure S13: Maximum likelihood of 119 genomes in Clade 1 *S. aureus* ST1 phylogeny (a) and temporal signal determined from TempEst 1.5.3 [15] (b). Phylogeny determined from 2414 variant sites. Initial root-to-tip divergence showed isolates within Clades 1.1, 1.2, 1.3 and 1.4 were deviating from the regression line. All isolates within Clade 1.2, 1.3 and 1.4 along with 66 isolates from Clade 1.1 were removed from further temporal analysis (Table S5). The phylogeny focused on 119 genomes with the reference strain H8195 labelled in pink **(a)**. The linear regression for the 119 genomes in the Clade 1 phylogeny had an estimated most recent common ancestor date of 1936 but a low R^2 (0.11) likely due to the narrow sampling date range and limited genetic diversity indicated by the low SNP count. The slope is determined based solely on the variant sites. The 95% confidence interval is shown in a grey dotted line (1834-1965) **(b)**.

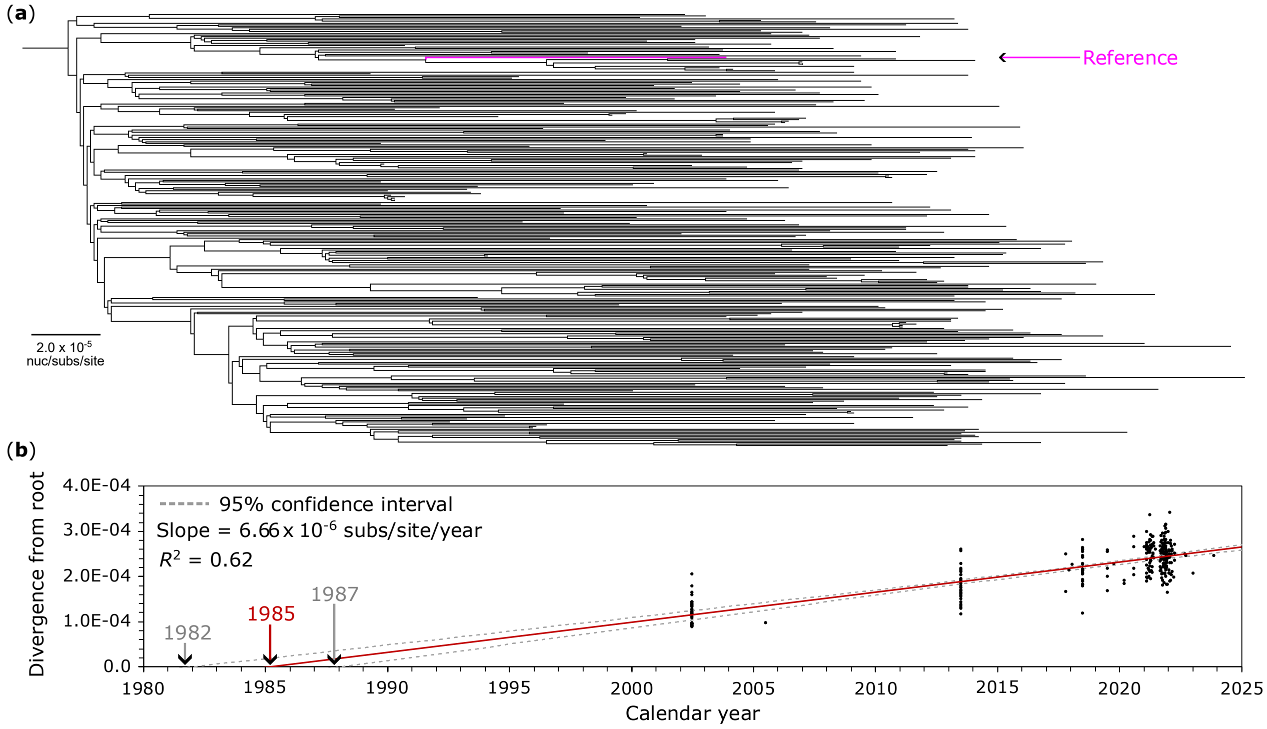

Figure S14: Maximum likelihood of 292 genomes in Clade 2 *S. aureus* ST1 phylogeny (a) and temporal signal determined from TempEst 1.5.3 [15] (b). Phylogeny determined from 16,554 variant sites. We removed 29 genomes that in initial root-to-tip divergence analysis were deviated from the regression line (Table S5). The reference strain 23EV612 is labelled in pink **(a)**. The temporal signal was investigated for the Clade 2 phylogeny. The linear regression for the 292 genome Clade 2 phylogeny with an estimated date of 1985 and a strong clock like signal R^2 (0.62). The slope is determined based solely on the variant sites. The 95% confidence interval is shown in a grey dotted line (1982-1987) **(b)**.

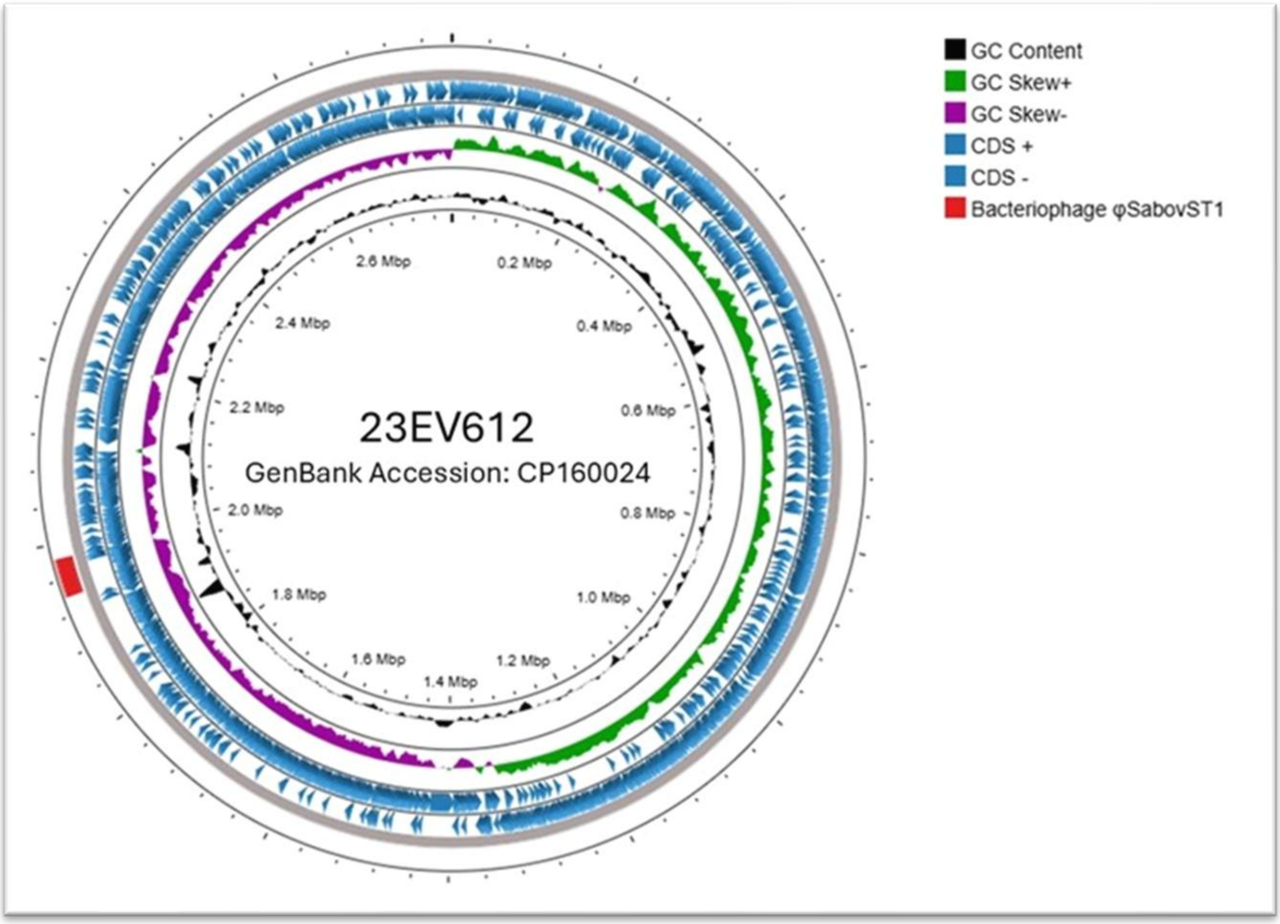

Figure S15: 23EV612 genome visualisation with bacteriophage φSabovST1 location. Annotation completed via Prokka and visualised in Proksee [27, 28]. The legend is displayed to the right, with the first inner circle being the positive GC skew and negative GC content, reverse strand coding sequence (CDS) genes and forward CDS genes, and the outer ring displays the location of the 44,213 bp phage genome determined by Phastest and PhageScope (https://phastest.ca, 25 June 2024 & https://phagescope.deepomics.org/, 9 July 2024).

References

1. Middleton J, Fox L, Pighetti G, Petersson-Wolfe C. Laboratory Handbook on Bovine Mastitis. 3rd ed. New Prague, MN USA: National Mastitis Council Inc 2017.

2. Boerlin P, Kuhnert P, Hussy D, Schaellibaum M. Methods for Identification of *Staphylococcus aureus* Isolates in Cases of Bovine Mastitis. J Clin Microbiol. 2003;41(2):767-71. doi:10.1128/JCM.41.2.767–771.2003

3. Oxford Nanopore Technologies. Guppy 6.0.1 [Internet]: Oxford Nanopore Technologies; 2022 [cited 2022 May 2]. Available from: https://community.nanoporetech.com/downloads.

4. Oxford Nanopore Technologies. Guppy 6.4.6 [Internet]: Oxford Nanopore Technologies; 2023 [cited 2023 May 6]. Available from: https://community.nanoporetech.com/downloads.

5. De Coster W, D'Hert S, Schultz DT, Cruts M, Van Broeckhoven C. NanoPack: visualizing and processing long-read sequencing data. Bioinformatics. 2018;34(15):2666-9. doi:10.1093/bioinformatics/bty149

6. Kolmogorov M, Yuan J, Lin Y, Pevzner PA. Assembly of long, error-prone reads using repeat graphs. Nat Biotechnol. 2019;37(5):540-6. doi:10.1038/s41587-019-0072-8

7. Hunt M, Silva ND, Otto TD, Parkhill J, Keane JA, Harris SR. Circlator: automated circularization of genome assemblies using long sequencing reads. Genome Biol. 2015;16(1):294. doi:10.1186/s13059-015-0849-0

8. Li H. Aligning sequence reads, clone sequences and assembly contigs with BWA-MEM. arXiv preprint arXiv:13033997. 2013

9. Danecek P, Bonfield JK, Liddle J, Marshall J, Ohan V, Pollard MO, et al. Twelve years of SAMtools and BCFtools. Gigascience. 2021;10(2). doi:10.1093/gigascience/giab008

10. Walker BJ, Abeel T, Shea T, Priest M, Abouelliel A, Sakthikumar S, et al. Pilon: An Integrated Tool for Comprehensive Microbial Variant Detection and Genome Assembly Improvement. PLoS One. 2014;9(11):e112963. doi:10.1371/journal.pone.0112963

11. Parks DH, Imelfort M, Skennerton CT, Hugenholtz P, Tyson GW. CheckM: assessing the quality of microbial genomes recovered from isolates, single cells, and metagenomes. Genome Res. 2015;25(7):1043-55. doi:10.1101/gr.186072.114

12. Gurevich A, Saveliev V, Vyahhi N, Tesler G. QUAST: quality assessment tool for genome assemblies. Bioinformatics. 2013;29(8):1072-5. doi:10.1093/bioinformatics/btt086

13. Alcock BP, Huynh W, Chalil R, Smith KW, Raphenya AR, Wlodarski MA, et al. CARD 2023: expanded curation, support for machine learning, and resistome prediction at the Comprehensive Antibiotic Resistance Database. Nucleic Acids Res. 2023;51(D1):D690-d9. doi:10.1093/nar/gkac920

14. Liu B, Zheng D, Zhou S, Chen L, Yang J. VFDB 2022: a general classification scheme for bacterial virulence factors. Nucleic Acids Res. 2022;50(D1):D912-d7. doi:10.1093/nar/gkab1107

15. Rambaut A, Lam TT, Max Carvalho L, Pybus OG. Exploring the temporal structure of heterochronous sequences using TempEst (formerly Path-O-Gen). Virus Evol. 2016;2(1):vew007. doi:10.1093/ve/vew007

16. Croucher NJ, Page AJ, Connor TR, Delaney AJ, Keane JA, Bentley SD, et al. Rapid phylogenetic analysis of large samples of recombinant bacterial whole genome sequences using Gubbins. Nucleic Acids Res. 2014;43(3):e15-e. doi:10.1093/nar/gku1196

17. BEAST Developers. Using TempEst for data exploration BEAST community 2025; [cited 2025 March 12]. Available from: https://beast.community/tempest_tutorial.

18. Bouckaert R, Heled J, Kuhnert D, Vaughan T, Wu CH, Xie D, et al. BEAST 2: a software platform for Bayesian evolutionary analysis. PLoS Comput Biol. 2014;10(4):e1003537. doi:10.1371/journal.pcbi.1003537

19. Bouckaert R, Vaughan TG, Barido-Sottani J, Duchene S, Fourment M, Gavryushkina A, et al. BEAST 2.5: An advanced software platform for Bayesian evolutionary analysis. PLoS Comput Biol. 2019;15(4):e1006650. doi:10.1371/journal.pcbi.1006650

20. Drummond AJ, Rambaut A, Shapiro B, Pybus OG. Bayesian Coalescent Inference of Past Population Dynamics from Molecular Sequences. Molecular Biology and Evolution. 2005;22(5):1185-92 .doi:10.1093/molbev/msi103

21. Drummond AJ, Ho SYW, Phillips MJ, Rambaut A. Relaxed Phylogenetics and Dating with Confidence. PLOS Biology. 2006;4(5):e88. doi:10.1371/journal.pbio.0040088

22. Darriba D, Taboada GL, Doallo R, Posada D. jModelTest 2: more models, new heuristics and parallel computing. Nature Methods. 2012;9(8):772-. doi:10.1038/nmeth.2109

23. Bianchini G, Sánchez-Baracaldo P. TreeViewer: Flexible, modular software to visualise and manipulate phylogenetic trees. Ecol Evol. 2024;14(2):e10873. doi:https://doi.org/10.1002/ece3.10873

24. ESRI. ArcGIS Pro 2023 [Internet]. ESRI, 2023 [cited 2023 Dec 4]. Available from: https://www.esri.com/en-us/arcgis/products/arcgis-pro/overview.

25. DairyNZ, LIC. New Zealand Dairy Statistics. New Zealand Dairy Statistics 2023-2024 [Internet]. New Zealand Dairy Statistics; 2024 [cited 2024 Dec 5]. Available from https://www.dairynz.co.nz/media/bywm13d4/dairy-statistics-2023-24.pdf2024

26. Kolde R. _pheatmap: Pretty Heatmaps: R package version 1.0.12. 2019. Available from: https://CRAN.R-project.org/package=pheatmap.

27. Seemann T. Prokka: rapid prokaryotic genome annotation. Bioinformatics. 2014;30(14):2068-9. doi:10.1093/bioinformatics/btu153

28. Grant JR, Enns E, Marinier E, Mandal A, Herman EK, Chen CY, et al. Proksee: in-depth characterization and visualization of bacterial genomes. Nucleic Acids Res. 2023;51(W1):W484-w92. doi:10.1093/nar/gkad326
